# Fully Well-Balanced Methods for Schwarzschild–Euler Equation in Gullstrand–Painlevé Coordinates

**DOI:** 10.1007/s10915-026-03324-6

**Published:** 2026-05-21

**Authors:** Ernesto Pimentel-García, Samuel Santos-Pérez, Isabel Cordero-Carrión, Carlos Parés

**Affiliations:** 1 Departamento de Matemática aplicada, Universidad de Málaga, C/ Dr. Ortiz Ramos s/n, 29071 Málaga, Spain; 2 Departamento de Matemáticas, Universitat de València, Dr. Moliner 50, 46100 Burjassot (Valencia), Spain; 3 Departamento de Análisis Matemático, Estadística e Investigación Operativa, y Matemática aplicada, Universidad de Málaga, Bulevar Louis Pasteur, 31, 29010 Málaga, Spain

**Keywords:** Well-balanced Methods, Euler Equation, Relativistic Fluids, General Relativity, 65M08, 65M12, 76-10, 76M12, 76Y05, 83C57, 83-08

## Abstract

We present a comprehensive formulation of the general-relativistic Euler equations in Gullstrand-Painlevé coordinates, providing a description of fluid dynamics that remains regular across the Schwarzschild event horizon. After establishing the system and examining its mathematical properties including well-posedness behaviour, Riemann invariants, and characteristic wave speeds, we obtain its stationary solutions in implicit form and analyze them in detail for a representative equation of state. Building on these results, we design high-order exactly well-balanced numerical schemes capable of preserving all stationary states of the system. Both first- and second-order methods are constructed. Extensive numerical experiments confirm the accuracy, robustness, and well-balanced behavior of the proposed schemes. The study offers both theoretical insight into relativistic fluid flows near black holes and practical tools for reliable long-term simulations in curved spacetimes.

## Introduction

The study of fluids in the strong gravitational fields of black holes (BHs) remains a cornerstone of modern theoretical and computational astrophysics. BHs, fundamental solutions of Einstein’s field equations [32, 34, 40, 49], provide a natural laboratory to explore the interaction between General Relativity (GR) and fluid dynamics. General Relativistic Hydrodynamics (GRHD) has emerged as a crucial tool for understanding astrophysical processes around compact objects [38, 43]. The dynamics of fluids near BHs influence the formation of accretion disks [7, 14, 42], relativistic jets [4, 6], and gravitational wave signals from binary mergers [5, 45]. In particular, the behavior of a perfect fluid freely falling toward a non-rotating (Schwarzschild) BH is a problem of great interest, with implications for accretion theory [1], BH astrophysics [44, 46], and high-energy phenomena such as gamma-ray bursts [36, 50] and active galactic nuclei [31, 35]. From a mathematical perspective, the problem of freely falling fluids in curved spacetime serves as a fundamental testbed for relativistic fluid equations, conservation laws, and shock wave formation under extreme conditions. Moreover, advances in Numerical Relativity have enabled increasingly precise simulations of fluid behavior in the presence of strong gravitational fields [3, 13, 21, 22, 29, 41], bridging the gap between theory and observation.

In this article, we analyze the motion of a perfect fluid undergoing radial free fall into a Schwarzschild BH. The analysis is carried out under the Cowling approximation, in which the central mass is held fixed and the background metric remains unaffected by accretion. Our focus lies on the theoretical formulation of the problem, examining fundamental aspects such as the velocity field, energy–momentum conservation, and the evolution of energy density and pressure in the relativistic regime. To this end, we employ the Gullstrand-Painlevé coordinates [37], which provide a convenient framework to describe the infalling motion. These coordinates cover the entire Schwarzschild spacetime in a single chart, unlike Schwarzschild coordinates, which break down at the event horizon. Other penetrating coordinates exist, such as the non-rotating Kerr–Schild and Eddington–Finkelstein coordinates; see [2, 48] for pedagogical introductions. The former contain two non-vanishing off-diagonal components in the metric tensor, while the latter employ a null coordinate instead of a time coordinate. In order to avoid introducing null coordinates (thereby preserving physical intuition) and to keep the number of non-zero off-diagonal metric components minimal, we consider Gullstrand–Painlevé coordinates a convenient starting point for working with penetrating coordinates in Schwarzschild spacetime.

This study not only provides insights into the fundamental physics of relativistic fluids but also contributes to our understanding of astrophysical scenarios where infalling matter plays a dominant role. To do so, we address the general-relativistic version of the Euler Equation in spherical symmetry using the mentioned coordinates, for which we find the analytic expressions of several families of stationary solutions. Although stationary solutions for this model already exist in Schwarzschild coordinates, see [25, 30], we go one step further by presenting a version in penetrating coordinates. However, we take the previous ones as a reference, as they will converge between each other at spatial infinity.

Due to the complexity of the generalized version of the Euler Equation, numerical methods are required to obtain its solutions. In this line, well-balanced methods for balanced laws [9] have beene been proven to be very powerful when stationary solutions exist. They are able to numerically recover these solutions, or their behavior when a perturbation is applied, with more precision than other schemes that treat the fluxes differently. To develop these methods is very convenient to have analytical expressions of the stationary solutions of the system of partial differential equations under resolution, or at least a numerical solution of it.

In [25] well-balanced methods were successfully applied to the discretization of the radial Euler Equation in the gravitational field of BHs expressed in Schwarzschild coordinates. The goal of this paper is to develop robust numerical tools able to simulate the free falling of radial perfect fluids towards a BH in the entire Schwarzschild spacetime by using the Gullstrand-Painlevé coordinates. The derivation of the model, its formulation as a system of balance laws, the analytical study of its stationary solutions, and the development of well-balanced finite-volume first- and second-order numerical methods are the milestones for achieving this goal.

In Section 2 we derive the general-relativistic Euler equations within the Gullstrand–Painlevé coordinate framework, establishing a formulation that remains regular across the Schwarzschild horizon. We examine the mathematical properties of the resulting system, including its well-posedness and the structure of its Riemann invariants and wave speeds. In Subsection 2.3 the stationary solutions are obtained in an implicit form, while Subsection 2.4 provides a detailed analysis of these solutions for a specific equation of state. This study allows us to develop high-order exactly fully well-balanced methods in Section 3 that are able to preserve all the stationary solutions of the system. Both first- and second-order schemes with this property are described. Section 4 then demonstrates the accuracy, robustness, and well-balanced behaviour of the proposed algorithms through a series of numerical experiments. Finally, Section 5 summarizes the main findings and outlines the broader implications of the work. Throughout the text we will use geometrized units, in which the speed of light in vacuum *c* and the universal gravitational constant *G* are set to 1.

## Euler Equation in Schwarzschild Spacetime

In classical physics, the conservation of energy and momentum is a fundamental principle governing the dynamics of physical systems. However, in the framework of GR, where spacetime is curved by the presence of mass and energy, the concept of energy-momentum conservation takes a more intricate form. Unlike in Special Relativity and Newtonian mechanics, where energy and momentum are globally conserved, GR introduces a local conservation law encoded in the energy-momentum tensor through the following covariant divergence equation:

1 $$\begin{aligned} \nabla _\mu T^{\mu \nu }=0, \end{aligned}$$

where $T^{\mu \nu }$ is the energy-momentum tensor field of the system. This expression ensures that energy and momentum are conserved in a differential sense within curved spacetime but does not necessarily imply a straightforward global conservation law due to the dynamical nature of spacetime itself.

In Newtonian mechanics, Euler Equation [24] governs the dynamics of the system, which at the same time represents momentum conservation. In GR the dynamics of a perfect fluid are described from Equation (1) considering the energy-momentum tensor of a perfect fluid, defined by:

2 $$\begin{aligned} T^{\alpha \beta } = (\mu +p)v^\alpha v^\beta + p \, g^{\alpha \beta }, \end{aligned}$$

where $\mu$ is the energy density, *p* is the pressure, $v^\alpha$ are the components of the Eulerian four-velocity of the fluid and $g^{\alpha \beta }$ the components of the metric tensor field. Energy and momentum conservation equations in GR are usually written as:

3 $$\begin{aligned}&\frac{d\mu }{d\tau }+(\mu +p)\,\nabla \cdot {\boldsymbol{v}}=0,\end{aligned}$$

4 $$\begin{aligned}&(\mu +p)\,\frac{D{\boldsymbol{v}}}{d\tau }+({\boldsymbol{g}} +{\boldsymbol{v}}\otimes {\boldsymbol{v}})\cdot \nabla p=0, \end{aligned}$$

where $\tau$ is the proper time of the fluid particle and $D{\boldsymbol{v}}/d\tau = {\boldsymbol{v}} \cdot \nabla {\boldsymbol{v}}$ is the covariant derivative along the wordline associated with ${\boldsymbol{v}}$. Equation (4) is a version of the Euler Equation in a curved spacetime that has an interesting resemblance to its classical version. However, it is also interesting to consider the equations derived directly from equations (1). This approach is used in [26], considering Schwarzschild coordinates $(t,r,\theta ,\phi )$. In these coordinates, the line element is

5 $$\begin{aligned} ds^2=-\left( 1-\frac{R}{r}\right) \,dt^2 + \left( 1-\frac{R}{r}\right) ^{-1} dr^2 + r^2\,(d\theta ^2 + \sin ^2\theta \, d\phi ^2), \end{aligned}$$

where *R* is the radius of the event horizon.

### Gullstrand-Painlevé Coordinates

Schwarzschild coordinates have a coordinate singularity at the event horizon. These types of singularities are not physical, in the sense that they have a pathology derived from the atlas used to cover the whole differential manifold. When using Schwarzschild coordinates, it is important to refrain from including this horizon in our numerical domain and imposing boundary conditions in its vicinity. However, the problem is removed directly when other coordinates are used, for example, the Gullstrand-Painlevé coordinates [37]. They can be obtained from Schwarzschild coordinates by the following change in the time coordinate:

6 $$\begin{aligned} T= t+ 2R\left( \sqrt{\frac{r}{R}}+\frac{1}{2}\log \left( \frac{\sqrt{r/R}-1}{\sqrt{r/R}+1}\right) \right) . \end{aligned}$$

In doing so, the line element takes the form:

7 $$\begin{aligned} ds^2=-dT^2+\left( \sqrt{\frac{R}{r}}\,dT+dr\right) ^2+r^2\,(d\theta ^2 + \sin ^2\theta \, d\phi ^2), \end{aligned}$$

where *T* is the Gullstrand-Painlevé time. Only the physical singularity at $r=0$ remains with this choice of coordinates. In order to write a fully developed expression for $(1)$, some Christoffel coefficients are needed. We list below those that are not zero:8We have bypassed those that can be derived from the symmetry . Now, we can write expression (1) with the energy-momentum tensor of a perfect fluid (2). We assume spherical flow, as in [26], i.e.,

$$\begin{aligned} {\boldsymbol{v}} ={\boldsymbol{v}} (T,r) = (v^0(T,r),\,v^1(T,r),0,0), \end{aligned}$$

where *T* and *r* are time and radial coordinates in Gullstrand-Painlevé coordinates. Let us denote $x^0=T$ and $x^1=r$. With this notation $v^0=dx^0/d\tau$ and $v^1=dx^1/d\tau$. In general, $v^1$ does not represent a velocity measured by an inertial observer. As such, it is not constrained by the speed of light and should be seen as a magnitude that encodes the radial flux of matter. However, $v^1$ can be interpreted as the velocity measured by a static observer located at spatial infinity, where the effects of gravity or spacetime curvature become negligible. Under this coordinate choice, Equation (1) transforms into the following two equations:

9 $$\begin{aligned} \begin{aligned}&\frac{\partial {\;\;}}{\partial {x^0}}\left( r^2(\mu +p)v^0v^0-r^2p\right) + \frac{\partial {\;\;}}{\partial {x^1}}\left( r^2(\mu +p)v^0v^1+r^2B\,p\right) \\&\;\;\;\;+\frac{rB}{2}\left( (\mu +p)( v^0 v^0 -1) -3p\right) = 0, \\&\frac{\partial {\;\;}}{\partial {x^0}}\left( r^2(\mu +p)v^0v^1+r^2B\,p\right) + \frac{\partial {\;\;}}{\partial {x^1}}\left( r^2(\mu + p ) v^1v^1 +r^2D p\right) \\&\;\;\;\; + \frac{R}{2}(\mu +p)+(R-2r)p = 0, \end{aligned} \end{aligned}$$

where $B=\sqrt{R/r}\ne 0$ and $D=1-R/r$. As the four-velocity is normalized by definition, $g_{\alpha \beta }v^\alpha v^\beta =-1$, only one of the two non-zero components of ${\boldsymbol{v}}$ is independent. Also, $v^0\ne 0$. Besides, an equation of state $p=p(\mu )$ is still needed. In the end, there are two equations and two independent variables (as it should be). Let us introduce the effective velocity,

10 $$\begin{aligned} v:=\frac{1}{B}\frac{v^1}{v^0}. \end{aligned}$$

Then, using the normalization condition, the following relations hold:

11 $$\begin{aligned} \begin{aligned} (v^0)^2&= \frac{r}{r-R(v+1)^2},\;\;\;\;\;\; (v^1)^2= \frac{R\,v^2}{r-R(v+1)^2}, \\ B v^0v^1&= \frac{R\, v}{r-R(v+1)^2}. \end{aligned} \end{aligned}$$

Finally, the previous system can be rewritten is a a more compact way:

12a $$\begin{aligned} \begin{aligned}&\frac{\partial {\;\;}}{\partial {x^0}}\left[ r^2\left( (\mu +p)\frac{r}{d}-p\right) \right] + \frac{\partial {\;\;}}{\partial {x^1}}\left[ r^2 B\left( (\mu +p)v \frac{r}{d}+p\right) \right] \\&\;\;\;\; + \frac{rB}{2}\left( (\mu +p)(1+v)^2\frac{R}{d}-3p\right) = 0, \end{aligned} \end{aligned}$$

12b $$\begin{aligned} \begin{aligned}&\frac{\partial {\;\;}}{\partial {x^0}}\left[ r^2 B\left( (\mu +p)v \frac{r}{d}+p\right) \right] + \frac{\partial {\;\;}}{\partial {x^1}}\left[ r^2\left( (\mu +p)v^2\frac{R}{d}+ Dp\right) \right] \\&\;\;\;\; + \frac{R}{2}(\mu +p)+(R-2r)p = 0, \end{aligned} \end{aligned}$$

where $d=r-R\,(u+1)^2$ is an effective distance. Let us introduce the variable

13 $$\begin{aligned} \nu =B(v+1)=B+\frac{v^1}{v^0}. \end{aligned}$$

With this choice, the following interesting compact relations hold:

14 $$\begin{aligned} v^0 = \frac{1}{\sqrt{1-\nu ^2}},\;\;\;\;\;\; v^1= \frac{\nu -B}{\sqrt{1-\nu ^2}}. \end{aligned}$$

It turns out that $\nu$ is the velocity measured by an inertial observer (whose wordline is a geodesic of spacetime). Indeed, let us consider the free-falling radial observer in Schwarzschild spacetime. The four-velocity of this observer is (see [37])

15 $$\begin{aligned} {\boldsymbol{u}} = (1,\,-\sqrt{R/r},0, 0). \end{aligned}$$

We can easily check that the time coordinate *T* is the proper time of this observer. The velocity of the fluid particle measured by this observer can be computed as (see [18])16 where is the projector over the reference space of the observer associated with ${\boldsymbol{u}}$, and $\varGamma$ is the Lorentz factor, from which a compact expression can be obtained:17On the other hand, as $\nu < 1$, it turns out that $d=r(1-\nu ^2)\ge 0$, so *d* can be correctly seen as a distance. Note that $\nu$ is the velocity of the fluid measured by a congruence of inertial (free-falling) observers, each located at a fixed radial coordinate *r*. Specifically, $\nu$ represents the relative velocity between the fluid particle placed at a fixed *r* and the local free-falling observer at that *r*. Consequently, its sign depends on whether the observer is moving faster ($\nu >0$) or slower ($\nu <0$) than the fluid element along their worldlines.

Working with $\nu$, we get aesthetically similar expressions to those of [26], where Schwarzschild coordinates are used. We can present () as the following system of nonlinear balance law:

18a $$\begin{aligned} \partial _0 V + \partial _1F(V,\,r)=S(V,\,r), \end{aligned}$$

where $$\partial _0 \equiv \partial \cdot /\partial x^0$$, $$\partial _1 \equiv \partial \cdot /\partial x^1$$ and

18b $$\begin{aligned} (1-\nu ^2) V = \begin{pmatrix} \mu +p\nu ^2 \\ \left( \mu +p\right) \nu - B\left( \mu +p\nu ^2\right) \end{pmatrix}, \end{aligned}$$

18c $$\begin{aligned} (1-\nu ^2)F = \begin{pmatrix} \left( \mu +p\right) \nu - B\left( \mu +p\nu ^2\right) \\ \left( \mu \nu ^2+p\right) - 2B\left( \mu +p\right) \nu + B^2\left( \mu +p\nu ^2\right) \end{pmatrix}, \end{aligned}$$

18d $$\begin{aligned} {(1-\nu ^2)S = \begin{pmatrix} -\frac{2}{r}\left( \mu +p\right) \nu + \frac{2B}{r}\left( \mu +p\nu ^2\right) - \frac{B}{2r}\left( (\mu +4p)\nu ^2-3p\right) \\ -\frac{2}{r}\left( \mu \nu ^2+p\right) + \frac{2B}{r}\left( \mu +p\right) \nu - \frac{2B^2}{r}\left( \mu +p\nu ^2\right) -\frac{B^2}{2r}(1-\nu ^2)\mu -\frac{3B^2-4}{2r}(1-\nu ^2) p \end{pmatrix}.} \end{aligned}$$

It has been assumed that we can get expressions of the primitive variables $(\mu , \nu )$ in terms of the conservative ones $V=(V^0,V^1)$, that is, $\mu =\mu (V)$ and $\nu =\nu (V)$. We denote by $p'(\mu )$ the derivative of the pressure *p* with respect to $\mu$. Looking at the Jacobian of the transformation, it can be checked that the recovery process is guaranteed if $[p'\nu ^4-2\nu ^3+(1-p')\nu ^2+1]\ne 0$ and $p\ne \mu$, which is always the case for $0<p'<1$ (a necessary physical condition, since the speed of sound has to always be lower than the speed of light). For example, if the sound speed is constant, say *k*, one has the equation of state $p=k^2\mu$. In that case, the following relations hold:

19 $$\begin{aligned} \nu =\frac{(1+k^2)-\sqrt{ (1+k^2)^2-4k^2\left( B+\frac{V^1}{V^0}\right) ^2 }}{2k^2\left( B+\frac{V^1}{V^0}\right) },\quad \mu =V^0 \left( \frac{1-\nu ^2}{1+k^2\nu ^2}\right) . \end{aligned}$$

Therefore, we assume that any state can be characterized by its vector of primitive variables,

$$\begin{aligned} W = \left[ \begin{array}{c} \mu \\ \nu \end{array} \right] . \end{aligned}$$

Equations (1.3d) in [26], which are analogous to those in (19), are recovered by imposing $B=0$. This makes sense since Schwarzschild and Gullstrand-Painlevé coordinates converge to Minkowski flat spacetime at spatial infinity ($r\rightarrow \infty$).

### Hyperbolicity

We are now determining the necessary and sufficient conditions that ensure hyperbolicity and genuine non-linearity properties of the system (). Let us consider its homogeneous part, $\partial _0 V+\partial _1 F(V,r_0)=0$, where the flux *F* is evaluated at a fixed radius $r_0$. The system can be written in terms of the Riemannn invariants and wave speeds:

20 $$\begin{aligned} \partial _0w_\pm +\lambda _\pm (w_\pm ,r_0)\partial _1 w_\pm =0. \end{aligned}$$

Considering the first Lemma of [26], we can proceed by following the outlined steps of its proof to compute the Riemann invariants and the wave speeds, respectively, as:

21 $$\begin{aligned} w_\pm =\frac{1}{2}\log \left( \frac{1+\nu }{1-\nu }\right) \pm \int _1^\mu \frac{\sqrt{p'(r)}}{x+p(r)}dx, \quad \lambda _\pm = \frac{\nu \pm \sqrt{p'(\mu )}}{1\pm \sqrt{p'(\mu )}\nu }-B. \end{aligned}$$

Again, the results in Schwarzschild coordinates are similar using the effective velocity $\nu$, and coincide at spatial infinity.

Following the usual nomenclature, a state $W = [\mu , \nu ]^T$ is said to be

- sonic if $$\lambda _+ \lambda _- = 0$$;
- subsonic if $$\lambda _+ \lambda _- < 0$$;
- supersonic if $$\lambda _+ \lambda - > 0$$.

According to (21), our system is: strictly hyperbolic if and only if $p'(\mu )>0, \; \forall \, \mu$; genuinely non-linear if and only if

22 $$\begin{aligned} 2p'(1-p')+p''(\mu +p)>0, \; \forall \, \mu ; \end{aligned}$$

and linearly degenerate if and only if the sign > of the previous inequality is replaced by an equal sign. This result is the same as using Schwarzschild coordinates (at it should be) and the proof is actually similar to the one presented in the first Proposition of [26]. The difference in this case is that, in the Gullstrand-Painlevé coordinates, we do not need to restrict our spatial domain to $r>R$.

In the particular case of an equation of state $p(\mu )=k^2\mu$, with *k* a constant value, the eigenvalues of the system are given by

23 $$\begin{aligned} \lambda _- = \frac{\nu -k}{1-k\,\nu }-B, \ \ \lambda _+ = \frac{\nu +k}{1+k\,\nu }-B. \end{aligned}$$

### Stationary Solutions

We derive the stationary solutions $\mu =\mu (r)$ and $\nu =\nu (r)$ of Equations (), for specific conditions at some $r=r_0$. There are significant differences in the derivation of these solutions compared to those obtained using Schwarzschild coordinates.

#### Lemma 1

If $\mu =\mu (r)$ and $\nu =\nu (r)$ are solutions to the system

24a $$\begin{aligned} \frac{dF(\mu , \nu , r)}{dr} = S(\mu , \nu , r), \end{aligned}$$

24b $$\begin{aligned} \mu (r_0)=\mu _0, \hspace{0.5cm} \nu (r_0)=\nu _0, \end{aligned}$$

and $\nu B \not =1$ (i.e. $\nu (r) \not = \sqrt{r/R}$), the following relations hold:

25a $$\begin{aligned}  &   r^2(1-B\nu )(\mu +p)\frac{\nu -B}{1-\nu ^2}=C_0,\end{aligned}$$

25b $$\begin{aligned}  &   \log |1-B\nu |-\frac{1}{2}\log |1-\nu ^2|+l(\mu )=D_0, \end{aligned}$$

where $l(\mu )$ is a primitive of $\frac{p'(\mu )}{\mu +p(\mu )}$ and $C_0$, $D_0$ are constants satisfying

26a $$\begin{aligned}  &   C_0=r_0^2(1-B_0\nu _0)(\mu _0+p(\mu _0))\frac{\nu _0-B_0}{1-\nu _0^2},\end{aligned}$$

26b $$\begin{aligned}  &   D_0=\log |1-B_0\nu _0|-\frac{1}{2}\log |1-\nu _0^2|+l(\mu _0), \end{aligned}$$

where $$B_0=\sqrt{R/r_0}$$.

#### Proof

The system (24a) can be written as

27a $$\begin{aligned} \frac{d(r^2F^0)}{dr}=- \frac{rB}{2}\left( (\mu +p)\frac{\nu ^2}{1-\nu ^2}-3p\right) , \end{aligned}$$

27b $$\begin{aligned} \frac{d(r^2F^1)}{dr} =- \frac{R}{2}(\mu +p)+r(D+1)p , \end{aligned}$$

where

28a $$\begin{aligned} F^0=(\mu +p) \frac{(\nu -B)}{1-\nu ^2}+B p, \end{aligned}$$

28b $$\begin{aligned} F^1= (\mu +p)\frac{(\nu -B)^2}{1-\nu ^2}+ Dp. \end{aligned}$$

We see that $F^1=(F^0-Bp)(\nu -B)+Dp$. Using this relation in Equation (27b), one can get

29 $$\begin{aligned} (\mu +p)\frac{(\nu -B)}{1-\nu ^2}\frac{d\nu }{dr}+(1-B\nu )\frac{dp}{dr}+\frac{B\nu }{2r}(\mu +p)=0. \end{aligned}$$

Since $-\frac{dB}{dr}=\frac{B}{2r}$, introducing the variable $\chi = B\nu$,

30 $$\begin{aligned} \frac{d\chi }{dr}-B\frac{d\nu }{dr}=\nu \frac{dB}{dr}=-\frac{B\nu }{2r}. \end{aligned}$$

Taking this relation into account in (29), and dividing by $(\mu +p)(1-\chi )$, we get

31 $$\begin{aligned} \frac{\nu }{1-\nu ^2}\frac{d\nu }{dr}+\frac{1}{\mu +p}\frac{dp}{dr}-\frac{1}{1-\chi }\frac{d\chi }{dr} =0, \end{aligned}$$

which can be integrated and (25b) is obtained. On the other hand, using the Equations (27a) and (29), one can see that

32 $$\begin{aligned} \frac{d\;\;}{dr}\left( r^2(1-B\nu )(\mu +p)\frac{\nu -B}{1-\nu ^2}\right) =0. \end{aligned}$$

Therefore, Equation (25a) can also be obtained. $\square$

It is interesting to note that the previous solutions can also be obtained from those derived in [26, 30] in Schwarzschild coordinates by a coordinate transformation. This is so because both times are proportional. Remember that for a four-vector a change of coordinates is expressed as

$$\begin{aligned} u^{\alpha '}=\frac{\partial {x^{\alpha '}}}{\partial {x^{\beta }}}u^{\beta }, \end{aligned}$$

where $\alpha '$ refers to the new coordinates and $\beta$ to the old ones. The unique component that varies in our case is the *T* component associated with the coordinate time of Gullstrand-Painlevé frame, which is expressed in terms of the *t* component associated with the coordinate time of Schwarzschild frame as

33 $$\begin{aligned} v^T=v^t+\frac{B}{1-B^2}v^r. \end{aligned}$$

When starting from the formulation in [26], the relation

34 $$\begin{aligned} \nu _{\text{ S }}=\frac{\nu -B}{1-B\nu } \end{aligned}$$

must be used, where

35 $$\begin{aligned} \nu _{\text{ S }}:=\frac{v^r}{(1-B^2)v^t} \end{aligned}$$

is the variable used in [26] to express the stationary solutions. On the other hand, Equation (25a) can be recovered from Equation (9) in [30], taking into account the second expression in (14).

Let us now look for stationary solutions satisfying $\nu \,B = 1$:

#### Lemma 2

Let us consider the function

$$\begin{aligned} \nu (r) = \sqrt{\frac{r}{R}}, \quad r \in (0, R). \end{aligned}$$

If $\mu (r)$ solves the differential equation

36 $$\begin{aligned} \frac{d\mu }{dr} = \frac{\mu + p}{2} \left( \frac{1}{R -r} - \frac{3}{r} \right) , \end{aligned}$$

then $\nu (r)$, $\mu (r)$ solve the system (24a).

#### Proof

It can be easily checked that, taking $B\,\nu = 1$, both (27a) and (27b) lead to the differential equation (36) for $\mu$. $\square$

The following result is a consequence of the implicit function theorem and guarantees the existence and uniqueness of the solutions provided some conditions.

#### Corollary 1

For all $(r,\,\mu ,\,\nu )=(r_0,\,\mu _0,\,\nu _0)$ satisfying the conditions

37 $$\begin{aligned} B\,\nu \ne 1,\;\;\nu ^2< 1,\;\; \nu \ne \frac{\sqrt{p'(\mu )}+ B}{B\sqrt{p'(\mu )}+ 1},\;\;\nu \ne \frac{\sqrt{p'(\mu )}- B}{B\sqrt{p'(\mu )}- 1}, \end{aligned}$$

there exist neighborhoods $I\subseteq \mathbb {R}$ of $r_0$ and $U\subseteq \mathbb {R}^{3}$ of $(r_0,\,\mu _0,\,\nu _0)$ such that $\forall r\in I, \exists !$ couple $(\mu ,\,\nu )$ such that the triplet $(r,\,\mu ,\,\nu )$ belongs to *U* and fulfill Equations (). Consequently, there exist a unique function

38 $$\begin{aligned} \begin{matrix} G &  : &  I &  \longrightarrow &  \mathbb {R }^2\\ &  &  r &  \mapsto &  (\mu (r),\,\nu (r)), \end{matrix} \end{aligned}$$

with $\left( r,\,\mu (r),\,\nu (r)\right)$ satisfying Equations (). Furthermore, $G\in \mathcal {C}^1(\mathring{I}\rightarrow \mathbb {R}^2)$.

The last two expressions in (37) establish sonic points in the forecoming Subsect. 2.4.

**Fig. 1:**
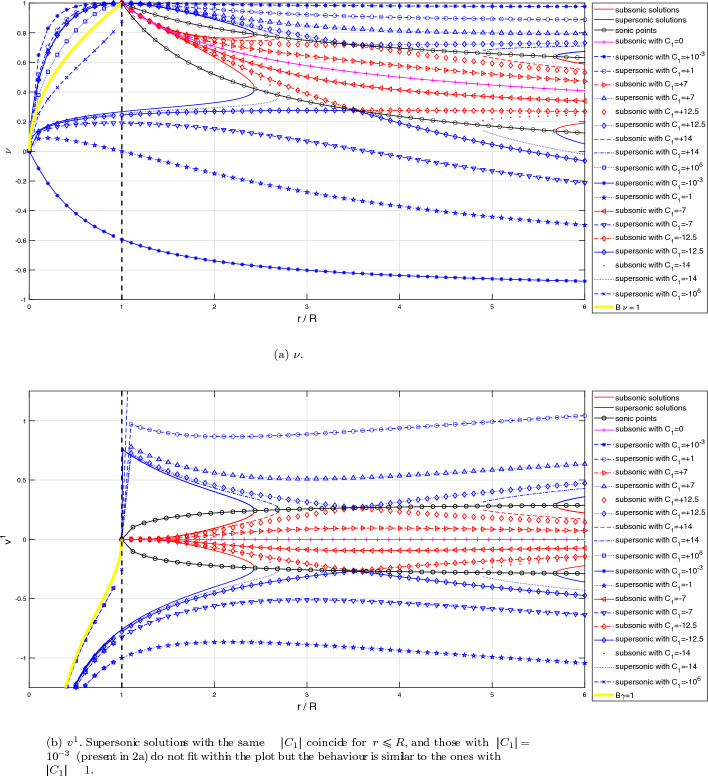
Some stationary solutions for different illustrative values of $C_1$ for $\nu$ (top) and $v^1$ (bottom)

As an example, let us compute some solutions for an equation of state $p(\mu )=k^2\mu$, with *k* a constant value. We have already stated that *k* plays the role of the speed of sound. The stationary solution to (24a), according to the previous Lemma, can be rewritten in the following form:

39 $$\begin{aligned} \begin{aligned}&r^2\text {sign}\left( 1-B\nu \right) \frac{\left( 1-\nu ^2\right) ^{\frac{1-k^2}{2k^2}}(\nu -B)}{\left| 1-B\nu \right| ^{1/k^2}}=C_1,\\  &\hspace{0.75cm} r^2\left( \frac{\nu -B}{1-\nu ^2}\right) \left( 1-B\nu \right) \mu =C_2, \end{aligned} \end{aligned}$$

where the constants $C_1,C_2\in \mathbb {R}$ depend on $r_0$, $\mu _0$ and $\nu _0$. From the definition $\nu =B(u+1)$, we can derive the corresponding expression for *u*. In Fig. 1 we show some solutions $v^1(r/R)$ and $\nu (r/R)$ for different values of the constant $C_1$. This figure is similar to the one obtained in [25] when $r\rightarrow \infty$. Note that $v^1$ does not play the role of any physical speed particle, but it is the radial component of the Eulerian velocity. We can check that inside the event horizon, $r<R$, the radial Eulerian velocity $v^1$ is negative, i.e points towards the center of the black hole. On the other hand, $|\nu |< 1$, which makes sense as it is a velocity measured by an inertial observer. Note that $|\nu |=1$ is discarded also looking at Equation (39) for $\mu$. However, we will consider these limit values for completeness from a mathematical point of view.

Observe that sonic states satisfy

$$ \nu = \frac{B \pm k}{1 \pm kB},$$

and this equality only involves the variables *r*, $\nu$. In Fig. 1 the graphs of the functions

$$ r \mapsto \frac{B - k}{1 - kB}, \quad r \mapsto \frac{B + k}{1 + kB}, $$

are also plotted (in black color). Sonic points of the stationary solutions correspond then to the intersections of the graph of $\nu$ with one of these two curves. Moreover, the regime is subsonic when the graph of $\nu$ lies between these two curves and supersonic otherwise (subsonic branches are plotted in red or magenta color and supersonic ones in blue). This graphical information will be useful in Subsection 2.4 to verify the regime of the stationary solutions based on the graph of $\nu$.

### Study of the Stationary Solutions for $p(\mu )=k^2 \mu$, with $0<k<1$

Let us consider first the stationary solutions satisfying $B\,\nu = 1$. In this case, the ordinary differential equation (ODE) (36) reduces to

$$ \frac{d\mu }{dr} = \frac{(1 + k^2)\mu }{2} \left( \frac{1}{R -r} - \frac{3}{r} \right) , $$

whose solutions are

40 $$\begin{aligned} \mu (r) = \frac{C}{\left[ r (R - r) \right] ^{\frac{1+ k^2}{2}}}. \end{aligned}$$

Therefore, according to Lemma 2, the pairs of functions

$$ \nu (r) = \sqrt{\frac{r}{R}}, \quad \mu (r) = \frac{C}{\left[ r (R - r) \right] ^{\frac{1+ k^2}{2}}}, \quad r \in (0,R), \quad C \in \mathbb {R},$$

constitute a family of stationary solutions. Observe that they all go to infinity in $r = 0$ and $r = R$ unless $C = 0$, i.e. unless $\mu \equiv 0$.

On the other hand, the stationary solutions such that $B\,\nu \not = 1$ are given by:

41 $$\begin{aligned} r^2\,\frac{\text {sign}(1-B\nu )\left( 1-\nu ^2\right) ^{\frac{1-k^2}{2k^2}}(\nu -B)}{|1-B\nu |^{1/k^2}}=C_1, \quad r^2\left( \frac{\nu -B}{1-\nu ^2}\right) \left( 1-B\nu \right) \mu =C_2, \end{aligned}$$

where $C_1$ and $C_2$ are given constants. Given a point *r*, $\nu (r)$ must satisfy the following equation in the variable $\nu$:

42 $$\begin{aligned} f(\nu ; B) = C_{1,r}, \end{aligned}$$

where $$C_{1,r} = \frac{C_{1}}{r^2}$$ and

43 $$\begin{aligned} f(\nu ; B) \equiv f(\nu ) = \frac{\text {sign}(1-B\nu )\left( 1-\nu ^2\right) ^{\frac{1-k^2}{2k^2}}(\nu -B)}{\left| 1-B\nu \right| ^{1/k^2}}. \end{aligned}$$

Once $\nu$ has been found by solving the equation (42), $\mu$ is given by

$$\begin{aligned} \mu = g(\nu , C_{2,r}), \end{aligned}$$

where $$C_{2,r} = \frac{C_{2}}{r^2}$$ and

$$\begin{aligned} g(\nu , C) = \frac{(1 - \nu ^2)\,C}{(\nu -B)(1 - B\nu )}. \end{aligned}$$

**Fig. 2:**
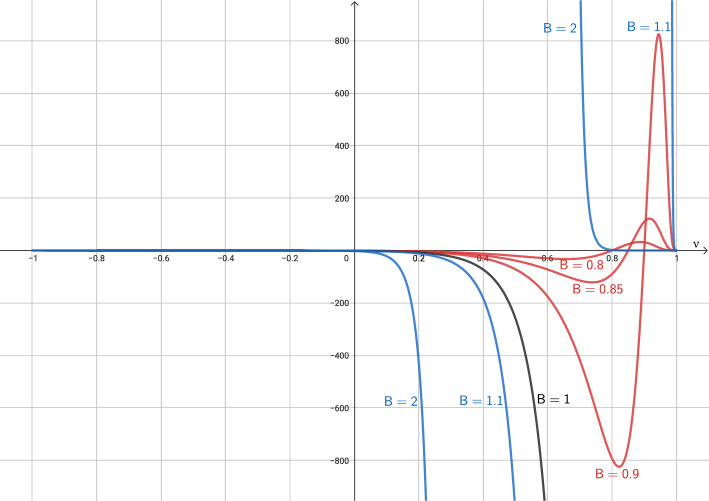
Graphic of function *f* for different values of *B*

In Fig. 2 we show the graphic of the function *f* for different values of *B*, to better understand the particular cases.

**Fig. 3:**
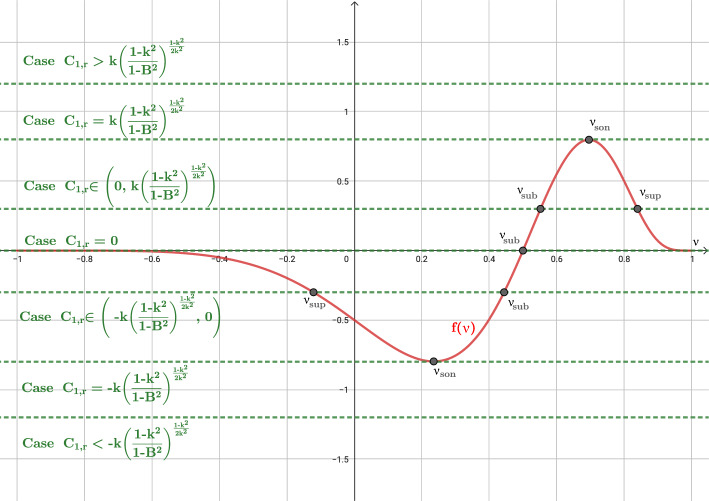
Graphic of function *f* in the case $B<1$. Different values of $C_{1,r}$ are shown. In particular $B=0.5$ is considered

**Fig. 4:**
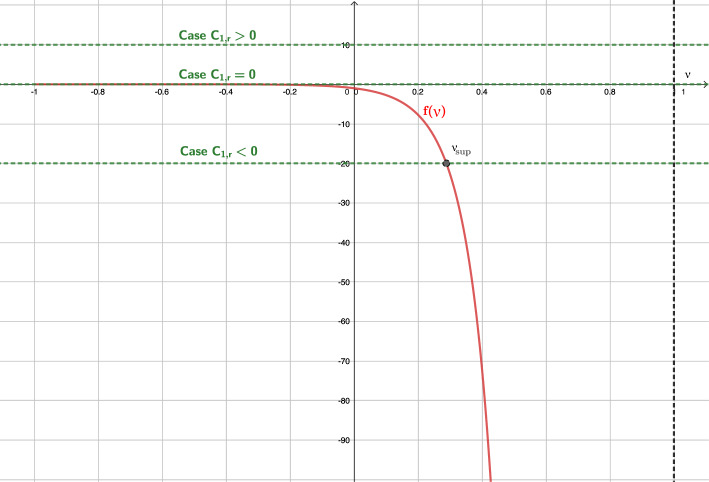
Graphic of function *f* in the case $B=1$. Different values of $C_{1,r}$ are shown

**Fig. 5:**
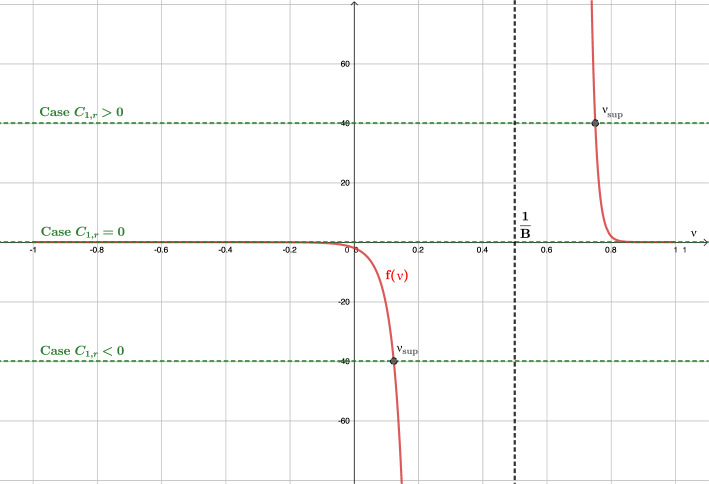
Graphic of function *f* in the case $B>1$. Different values of $C_{1,r}$ are shown. In particular $B=2$ is considered

After an exhaustive study of (43), we can distinguish the following three cases:

1. $B<1$, i.e., outside the event horizon. In this case (43) reads:
  44 $$\begin{aligned} f(\nu ) = (\nu -B)\frac{\left( 1-\nu ^2\right) ^{\frac{1-k^2}{2k^2}}}{\left( 1-B\nu \right) ^{1/k^2}}. \end{aligned}$$
  In Fig. 3 we plot the graphic of *f* for a given value of *B*, and we observe 4 possible cases for the roots of (42) depending on the value of $C_{1,r}$:
  - $C_{1,r}\in \Bigg (0, k\left( \frac{1-k^2}{1-B^2}\right) ^{\frac{1-k^2}{2k^2}}\Bigg )$. There are two roots:
    $$\begin{aligned} \nu _{sub}\in \left( B, \frac{k+B}{kB+1}\right) , \ \ \nu _{sup}\in \left( \frac{k+B}{kB+1},1\right) , \end{aligned}$$
    such that the states
    $$W_{sub} = \left[ \begin{array}{c} \mu _{sub} \\ \nu _{sub} \end{array}\right] , \quad W_{sup} = \left[ \begin{array}{c} \mu _{sup} \\ \nu _{sup} \end{array}\right] ,$$
    where $\mu _{sub}=g(\nu _{sub}, C_{2,r})$ and $\mu _{sup}=g(\nu _{sup}, C_{2,r})$, are subsonic and supersonic, respectively.
  - $C_{1,r} = 0$. There is only one root $\nu = B$ whose corresponding state is subsonic. From a mathematical point of view, the limit solutions $|\nu |=1$ can be also included in this case.
  - $C_{1,r}\in \Bigg ( -k\left( \frac{1-k^2}{1-B^2}\right) ^{\frac{1-k^2}{2k^2}}, 0\Bigg )$. There are two roots:
    $$\begin{aligned} \nu _{sub}\in \left( \frac{k-B}{kB-1}, B\right) , \ \ \nu _{sup}\in \left( -1,\frac{k-B}{kB-1}\right) , \end{aligned}$$
    such that the corresponding states $W_{sub}$ and $W_{sup}$ are subsonic and supersonic, respectively.
  - $|C_{1,r}| = k\left( \frac{1-k^2}{1-B^2}\right) ^{\frac{1-k^2}{2k^2}}$. There is only one root:
    $$\nu _{son} = {\left\{ \begin{array}{ll} \displaystyle \frac{k-B}{kB-1}, &  \text {if } C_{1,r} = -k\left( \frac{1-k^2}{1-B^2}\right) ^{\frac{1-k^2}{2k^2}}, \\ \displaystyle \frac{k+B}{kB+1}, &  \text {if } C_{1,r} = k\left( \frac{1-k^2}{1-B^2}\right) ^{\frac{1-k^2}{2k^2}}, \end{array}\right. }$$
    whose corresponding state $W_{son} = [\mu _{son}, \nu _{son}]^T$, where $\mu _{son}= g(\nu _{son}, C_{2,r})$, is sonic.
  - $|C_{1,r}| > k\left( \frac{1-k^2}{1-B^2}\right) ^{\frac{1-k^2}{2k^2}}$. There is no root.
2. $B=1$, i.e., at the event horizon. In this case $(1-B\,\nu )=(1-\nu )>0$ and (43) reduces to:
  45 $$\begin{aligned} f(\nu ) = -\left( \frac{1+\nu }{1-\nu }\right) ^{\frac{1-k^2}{2k^2}}. \end{aligned}$$
  In Fig. 4 we plot the graphic of *f*, and we observe 3 possible cases for the roots of (42) depending on the value of $C_{1,r}$:
  - $C_{1,r} > 0$. There is no solution. From Fig. 1a, we observe that the limit value $\nu =1$, physically excluded, is reached for all the cases $C_1>0$.
  - $C_{1,r} = 0$. There is a unique root $\nu = -1$. Nevertheless, given any constant $C_2$ no $\mu$ can be chosen so that the second equation in (41), which in this case boils down to
    $$\begin{aligned} r^2 \frac{\nu -1}{\nu + 1} \mu = C_2, \end{aligned}$$
    is satisfied. Therefore no stationary solution can reach the value $\nu =-1$ at $B= 1$. From Fig. 2, we can understand that the limit for $B=1$ close to $\nu =1$ results into a vertical line at this value for the graphic of *f*, and therefore *f* is not anymore a function (it is not single valued). From Fig. 1a, we observe that the limit value $\nu =1$, physically excluded, is reached for the case $C_1=0$. Also, from a mathematical point of view, the limit values $\nu =\pm 1$, physically excluded, are reached for the curves with constant values $\nu =\pm 1$, respectively.
  - $C_{1,r} < 0$. There is a unique root $\nu _{sup} \in (-1,1)$ given by:
    $$\begin{aligned} \nu _{sup} = \frac{A-1}{A+1}, \end{aligned}$$
    where $A = \left( -C_{1,r}\right) ^{\frac{2k^2}{1-k^2}}$. The corresponding state $W_{sup}$ is supersonic.
3. $B>1$, i.e., inside the event horizon. In this case (43) reads:
  46 $$\begin{aligned} f(\nu ) = \frac{\text {sign}(1-B\nu )\left( 1-\nu ^2\right) ^{\frac{1-k^2}{2k^2}}(\nu -B)}{|1-B\nu |^{1/k^2}}. \end{aligned}$$
  In Fig. 5 we plot the graphic of *f* for a given value of *B*, and we observe 3 possible cases for the roots of (42) depending on the value of $C_{1,r}$:
  - $C_{1,r}>0$. There is a unique root $\nu _{sup}\in \left( \frac{1}{B}, 1\right)$ whose corresponding state $W_{sup}$ is supersonic.
  - $C_{1,r}=0$. There are two roots $\nu = \pm 1$ but, again, no value for $\mu$ can be chosen so that the second equation in (41) is satisfied. Therefore, no stationary solution can reach the values $\pm 1$ at $B > 1$.
  - $C_{1,r}<0$. There is a unique root $\nu _{sup}\in (-1, \frac{1}{B})$ whose corresponding state $W_{sup}$ is supersonic.

**Fig. 6:**
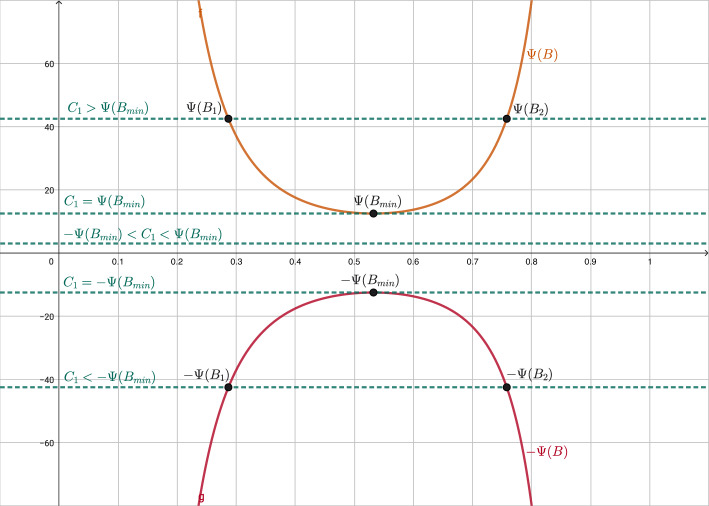
Graphic of function $\varPsi (B)$ with different values of $C_1$

Let us discuss the range of definition of the stationary solutions of the system:

- According to Case 1 ($B<1$), a stationary solution verifying (41) with $C_1>0$ can only be defined in the interval $r \in (R, \infty )$ if
  $$\begin{aligned} C_1 \le k\,r^2 \left( \frac{1-k^2}{1-B^2}\right) ^{\frac{1-k^2}{2k^2}} = k\,\frac{R^2}{B^4} \left( \frac{1-k^2}{1-B^2}\right) ^{\frac{1-k^2}{2k^2}}, \quad \forall B \in (0,1). \end{aligned}$$
  Analogously, a stationary solution with $C_1 < 0$ can only be defined in the interval $r \in (R, \infty )$ if
  $$\begin{aligned} -C_1 \le k\frac{R^2}{B^4} \left( \frac{1-k^2}{1-B^2}\right) ^{\frac{1-k^2}{2k^2}}, \quad \forall B \in (0,1). \end{aligned}$$
  On the other hand, it can be checked that the function
  47 $$\begin{aligned} B \in (0,1) \rightarrow \varPsi (B) := k\frac{R^2}{B^4} \left( \frac{1-k^2}{1-B^2}\right) ^{\frac{1-k^2}{2k^2}} \end{aligned}$$
  reaches its minimum at
  $$\begin{aligned} B_{min} = \frac{2}{\sqrt{3 + \frac{1}{k^2}}}. \end{aligned}$$
  In Fig. 6 the graphic of function $\varPsi (B)$ is plotted.
  For $C_1 = 0$ it also exists a subsonic stationary solution in the interval $r\in (R,\infty )$ given by $\nu = B$.
  Therefore, global stationary solutions outside the horizon, i.e., solutions that can be defined in the interval $r \in (R,\infty )$, can be built only if
  $$ C_1 \in \left[ -\varPsi (B_{min}),\varPsi (B_{min}) \right] . $$
  For the values in the interior of this interval, $|C_1|< \varPsi (B_{min})$, the stationary solution is always supersonic or subsonic. Moreover, for the values at the boundaries of this interval, $C_1=\pm \varPsi (B_{min})$, the stationary solutions are supersonic / subsonic in an interval $r\in (R,\tilde{R})$, with $\tilde{R}=R/B_{min}^2=R (3+1/k^2)/4$, then they cross the line of the sonic points with a smooth derivative at the radius $r=\tilde{R}$, continuing with subsonic / supersonic values, respectively, in the interval $r\in (\tilde{R},\infty )$. The expression for $\tilde{R}$ will be the same in the next paragraphs. In Fig. 1a  these curves correspond to the values $C_1=\pm 12.50$.
  For values of $C_1$ outside this range, the equation
  $$ \varPsi (B) = C_1 $$
  has two roots $B_1 \le B_2$ in the interval $B \in (0,1)$ (see Fig. 6). 4 non-global stationary solutions corresponding to $C_1$ can then be built:
  (i) One supersonic and one subsonic stationary solutions defined in the interval $r\in (R/B_1^2, \infty )$ that reach the same sonic state at $r = R/B_1^2$ with infinite derivative.
  (ii) One supersonic and one subsonic stationary solutions defined in the interval $r\in (R, R/B_2^2)$ that reach the same sonic state at $r = R/B_2^2$ with infinite derivative.
  These stationary solutions can be seen in Fig. 1a: they correspond to branches that have a return point located at the line formed by the critical sonic states (black circles and black line). In this case, these curves cross the line of sonic points with infinite derivative, contrary to the solutions with $|C_1|=\varPsi (B_{min})$.
- According to Case 3 ($B>1$) only supersonic stationary solutions can be built in the interval $r \in (0, R)$ for any value of $C_1\ne 0$.

**Fig. 7:**
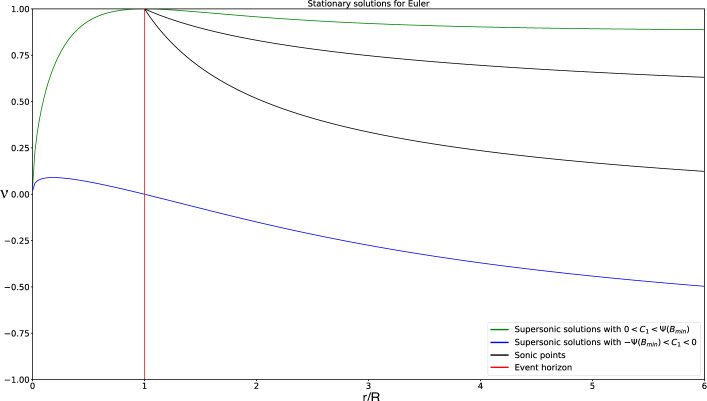
Graphic of global smooth supersonic stationary solutions

#### Global Continuous Stationary Solutions

A careful analysis, detailed in the three results presented in Appendix A, allows us to see under what circumstances these stationary solutions defined in (0, *R*) and $(R, \infty )$ can be connected continuously or differentially at $r = R$. According to this analysis, only 7 families of continuous global stationary solutions can be built:

**Fig. 8:**
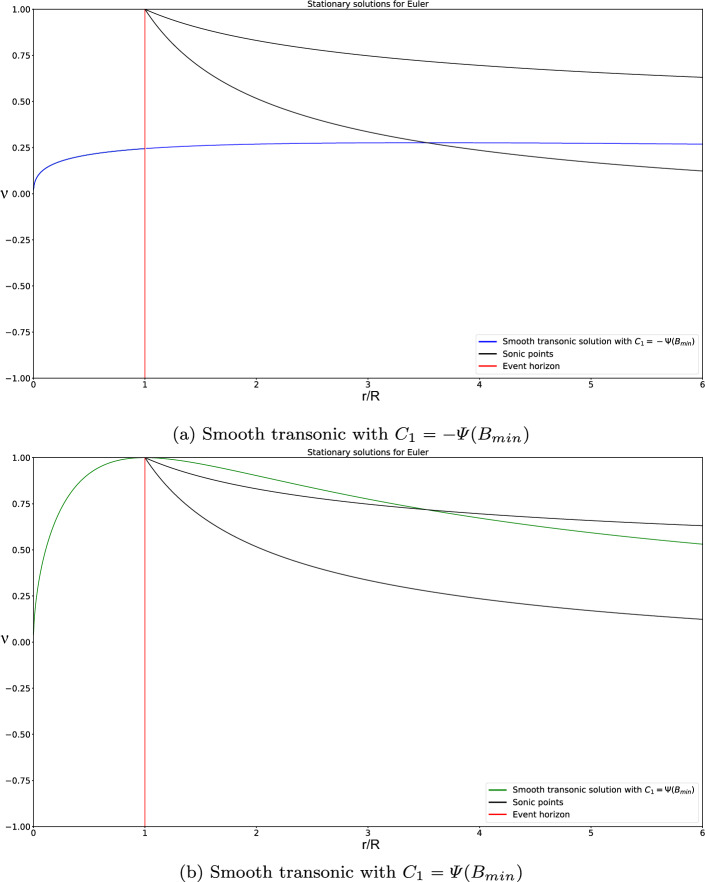
Graphic of global smooth transonic stationary solutions

**Fig. 9:**
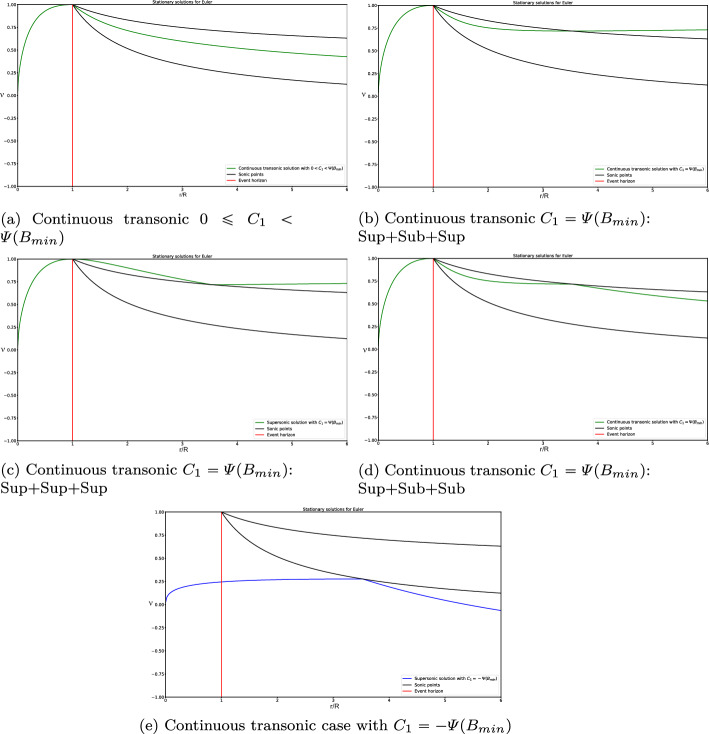
Graphic of global continuous transonic stationary solutions

1. Smooth supersonic stationary solutions corresponding to
  $$\begin{aligned} C_1 \in \left( -\varPsi (B_{min}), 0 \right] . \end{aligned}$$
  See an illustrative example in Fig. 7. In the particular case $C_1 = 0$, we have the non-physical solution $\nu \equiv -1$.
2. Smooth transonic stationary solution, supersonic in the interval $r\in (0,\tilde{R})$, sonic at the radius $r=\tilde{R}$, and subsonic in the interval $r\in (\tilde{R},\infty )$, corresponding to
  $$\begin{aligned} C_1 = - \varPsi (B_{min}). \end{aligned}$$
  See an illustrative example in Fig. 8a.
3. Smooth stationary solutions corresponding to
  $$\begin{aligned} C_1 \in \left[ 0, \varPsi (B_{min})\right) \end{aligned}$$
  that are supersonic everywhere but at $r = R$, where they take the sonic value 1 that is not physically allowed. The variable $V^0$ only remains bounded if $C_2 = 0$, in which case $\mu \equiv V^0 \equiv V^1 \equiv 0$. In the particular case $C_1 = 0$ we have the non-physical solution $\nu \equiv 1$. See an illustrative example in Fig. 7.
4. Smooth transonic stationary solution, supersonic in the interval $r\in (0,\tilde{R})$, sonic at the radius $r=\tilde{R}$, and subsonic in the interval $r\in (\tilde{R},\infty )$, corresponding to
  $$\begin{aligned} C_1 = \varPsi (B_{min}). \end{aligned}$$
  Again, $\nu$ takes the non-physical value 1 at $r = R$ and the variable $V^0$ only remains bounded if $C_2 = 0$, in which case $\mu \equiv V^0 \equiv V^1 \equiv 0$. See an illustrative example in Fig. 8b.
5. Continuous transonic stationary solutions, supersonic in the interval $r\in (0,R)$ and subsonic in the interval $r\in (R,\infty )$, corresponding to
  $$\begin{aligned} C_1\in \left( 0, \varPsi (B_{min})\right) . \end{aligned}$$
  See an illustrative example in Fig. 9a. As in the previous case, $\nu$ is not differentiable at $r = R$, where it takes the non-physical value 1, and the variable $V^0$ only remains bounded if $C_2 = 0$, in which case $\mu \equiv V^0 \equiv V^1 \equiv 0$.
6. Three continuous stationary solutions corresponding to
  $$\begin{aligned} C_1 = \varPsi (B_{min}) \end{aligned}$$
  that are not differentiable at some points:
  The first one is supersonic in the interval $r\in (0,R)$, sonic at $r=R$, subsonic in the interval $r\in (R,\tilde{R})$, sonic at the radius $r=\tilde{R}$, and supersonic in the interval $r\in (\tilde{R},\infty )$. This solution is not differentiable at $r = R$. See an illustrative example in Fig. 9b.
  The second one is supersonic in the interval $r\in (0,R)$, sonic at $r=R$, supersonic in the interval $r\in (R,\tilde{R})$, sonic at the radius $r=\tilde{R}$, and supersonic in the interval $r\in (\tilde{R},\infty )$. This solution is not differentiable at $r = R$ and $r = \tilde{R}$. See an illustrative example in Fig. 9c.
  The last one is supersonic in the interval $r\in (0,R)$, sonic at $r=R$, subsonic in the interval $r\in (R,\tilde{R})$, sonic at the radius $r=\tilde{R}$, and subsonic in the interval $r\in (\tilde{R},\infty )$. This solution is not differentiable at $r = R$ and $r = \tilde{R}$. See an illustrative example in Fig. 9d.
  These three solutions share the same branch in the interval $r\in (0,R)$ but follow different ones from this radius on. Also, $\nu$ takes the non-physical value 1 at $r=R$. Again, the variable $V^0$ only remains bounded if $C_2 = 0$, in which case $\mu \equiv V^0 \equiv V^1 \equiv 0$.
7. Finally, a continuous stationary solution corresponding to
  $$\begin{aligned} C_1 = -\varPsi (B_{min}) \end{aligned}$$
  which is supersonic in the interval $r\in (0,\tilde{R})$, sonic at $r=\tilde{R}$, and supersonic in the interval $r\in (\tilde{R},\infty )$. This solution is not differentiable at $r = \tilde{R}$. See an illustrative example in Fig. 9e.

## High-Order Exactly Fully Well-Balanced Scheme

We recall system ():

48 $$\begin{aligned} \partial _t V + \partial _r F(V,\,r)=S(V,\,r), \end{aligned}$$

where *V*, *F* and *S* are given by (18b), (18c) and (18d), respectively. We are interested in the preservation of the stationary solutions of system (48), i.e., the solutions that satisfy:

49 $$\begin{aligned} \partial _r F(V,\,r)=S(V,\,r), \end{aligned}$$

that have been studied in Subsection 2.3. Regarding the numerical schemes, let us consider uniform meshes of length $\varDelta r$, consisting on cell $I_{i} = [r_{i-\frac{1}{2}}, r_{i+\frac{1}{2}}]$, where $r_{i}$ represents the midpoint of each cell.

Following [9, 33] we consider the following semi-discrete finite-volume methods:

50 $$\begin{aligned} \overline{V}_i'(t) = -\frac{1}{\varDelta r} \left( F_{i+\frac{1}{2}} - F_{i-\frac{1}{2}} - \int _{r_{i-\frac{1}{2}}}^{r_{i+\frac{1}{2}}} S(P^t_i(r),r) \, dr \right) , \end{aligned}$$

where:

- $\overline{V}_i(t)$ is the approximation provided by the numerical method of the average of the exact solution in the $i$-th cell, $[r_{i-\frac{1}{2}}, r_{i+\frac{1}{2}}]$, at time $t$, i.e.,
  51 $$\begin{aligned} \overline{V}_i(t) \approx \frac{1}{\varDelta r} \int _{r_{i-\frac{1}{2}}}^{r_{i+\frac{1}{2}}} V(r, t) \, dr; \end{aligned}$$
- $P^t_i(r)$ is the approximation of the solution in the $i$-th cell given by a high-order reconstruction operator applied to the sequence of cell averages $\{\overline{V}_j(t)\}$, i.e.,
  52 $$\begin{aligned} P^t_i(r) = P_i(r; \{\overline{V}_j(t)\}_{j \in S_i}), \end{aligned}$$
  where $S_i$ represents the set of indexes of the cells belonging to the stencil of the *i*th cell;
- $$F_{i+\frac{1}{2}} = \mathbb {F}(V^{t,-}_{i+\frac{1}{2}}, V^{t,+}_{i+\frac{1}{2}})$$, where
  $$ V^{t,-}_{i+\frac{1}{2}} = P^t_i(r_{i+\frac{1}{2}}), \quad V^{t,+}_{i+\frac{1}{2}} = P^t_{i+1}(r_{i+\frac{1}{2}}), $$
  are the reconstructed states at the intercells and $\mathbb {F}$ is a consistent numerical flux.

The reconstruction operator will be selected so that the numerical method has the well-balanced property set out in the following definition:

### Definition 1

The numerical method (50) is said to be exactly fully well-balanced (EFWB) if the sequence of cell averages $\{\overline{V}^*_i\}$ of any continuous stationary solution $V^*$ satisfying (49) is an equilibrium of the ODE (50).

The ODE (50) can be formally written as

53 $$\begin{aligned} \overline{V}_i'(t) = L_i(\{\overline{V}_j(t) \}; \varDelta x) \end{aligned}$$

so that, for EFWB methods one has

$$ L_i(\{\overline{V}^*_j \}; \varDelta x) = 0, \quad \forall i, $$

for all the sequences of cell averages $\{\overline{V}^*_i\}$ of stationary solutions of the system.

In order to obtain a fully discrete method, a one-step ODE solver will be applied to (53):

54 $$\begin{aligned} \overline{V}_i^{k+1} = H_i(\{\overline{V}_j^k \}; \varDelta x, \varDelta t_k), \end{aligned}$$

where $\varDelta t_k$ is the time step. The EFWB of the fully discrete method is preserved provided that for every *i*

55 $$\begin{aligned} L_i(\{\overline{V}^*_j \}; \varDelta x) = 0 \implies H_i(\{\overline{V}^*_j \}; \varDelta x, \varDelta t_k) = \overline{V}^*_i, \end{aligned}$$

what is true for every Runge-Kutta method, as the TVD RK methods used here.

It can be easily verified that (50) is exactly fully well-balanced if the reconstruction operator is exactly fully well-balanced according to the following definition (see [9] for details):

### Definition 2

The reconstruction operator $\{P_i(r)\}$ is said to be exactly fully well-balanced if, when applied to the sequence of cell averages $\{\overline{V}^*_i\}$ of any continuous stationary solution $V^*$ satisfying (49), one has

$$ P_i(r) = V^*(r), \quad \forall r \in [r_{i-\frac{1}{2}}, r_{i+\frac{1}{2}}], \quad \forall i, $$

$$ V^\pm _{i+\frac{1}{2}} = V^*(r^\pm _{i+\frac{1}{2}}). $$

### Exactly Fully Well-Balanced Reconstruction Operator

Standard reconstruction operators are not well-balanced in general. Following [8, 9], in order to design an exactly fully well-balanced reconstruction operator, we first need to select a standard high-order reconstruction operator that we denote by:

$$\begin{aligned} Q_{i}(x; \{\overline{V}_{j}\}_{j\in S_{i}}), \end{aligned}$$

which provides a high-order approximation of the solution at the cells: some examples are MUSCL [47], ENO [19], WENO [23, 28], CWENO [11, 12, 27], etc. Once this standard operator has been chosen, it is modified to become well-balanced as follows: given a family of cell averages $\{\overline{V}_i\}$, to compute the reconstruction $P_i$ at the cell $[r_{i-\frac{1}{2}}, r_{i+\frac{1}{2}}]$:

1. Find, if possible, the stationary solution $V_{i}^{*}(r)$ such that:
  56 $$\begin{aligned} \frac{1}{\varDelta r}\int _{r_{i-\frac{1}{2}}}^{r_{i-\frac{1}{2}}} V_{i}^{*}(r)dr = \overline{V}_{i}. \end{aligned}$$
  In other case define $V_{i}^{*}\equiv \overline{V}_{i}^{n}$.
2. Compute the fluctuations $\{\overline{W}_{j}\}_{j\in S_{i}}$ as follows:
  57 $$\begin{aligned} \overline{W}_{j} = \overline{V}_{j} - \frac{1}{\varDelta r} \int _{r_{j-\frac{1}{2}}}^{r_{j+\frac{1}{2}}}V_{i}^{*}(r)dr, \ \ j\in S_{i}. \end{aligned}$$
3. Apply the standard reconstruction operator to the fluctuations $\{\overline{W}_{j}\}_{j\in S_{i}}$:
  $$ Q_{i}(r) = Q_{i}(r; \{\overline{W}_{j}\}_{j\in S_{i}}). $$
4. Defining the modified reconstruction as follows:
  58 $$\begin{aligned} P_{i}(r) = V_{i}^{*}(r) + Q_{i}(r). \end{aligned}$$

It can be clearly seen that, if $\{ \overline{V}_j \}$ are the cell averages of a stationary solution $V^*$, then $\overline{W}_j = 0, \,\forall j \in S_i$, and thus $P_i(r) = V^*(r), \forall r \in [r_{i-1/2}, r_{i + 1/2}]$.

#### Remark 1

$V_{i}^{*}(r)$ is a local stationary solution obtained in the cell *i*, satisfying (56). This local strategy also ensures the preservation of global stationary solutions $V^{*}(r)$ when they exist.

### Quadrature Formula

When a quadrature formula,

59 $$\begin{aligned} \int _{r_{i-\frac{1}{2}}}^{r_{i+\frac{1}{2}}} f(r) dr \approx \varDelta r \sum _{m=1}^{M} \beta _{m}f(r_{i}^{m}), \end{aligned}$$

is used to compute the integral of the source term, the well-balanced property of the scheme may be lost. Therefore, following [9], we must replace (56) and (57) by:

1. Find, if possible, the stationary solution $V_{i}^{*}(r)$ such that:
  60 $$\begin{aligned} \sum _{m=1}^{M} \beta _{m}V_{i}^{*}(r_{i}^{m}) = \overline{V}_{i}. \end{aligned}$$
  In other case, define $V_{i}^{*}\equiv \overline{V}_{i}^{n}$.
2. Compute the fluctuations $\{\overline{W}_{j}\}_{j\in S_{i}}$ as follows:
  61 $$\begin{aligned} \overline{W}_{j} = \overline{V}_{j} - \sum _{m=1}^{M} \beta _{m} V_{i}^{*}(r_{j}^{m}), \ \ j\in S_{i}. \end{aligned}$$

Moreover, we rewrite our scheme (50) in the equivalent form:

62 $$\begin{aligned} \overline{V}_i'(t) = -\frac{1}{\varDelta r} \Bigg ( F_{i+\frac{1}{2}}-F(V_{i}^{t,*}(r_{i+\frac{1}{2}}), r_{i+\frac{1}{2}})+F(V_{i}^{t,*}(r_{i-\frac{1}{2}}),r_{i-\frac{1}{2}}) - F_{i-\frac{1}{2}} \nonumber \\ - \int _{r_{i-\frac{1}{2}}}^{r_{i+\frac{1}{2}}} \big [S(P^t_i(r),r)-S(V^{t,*}(r), r)\big ] \, dr \Bigg ), \;\; \end{aligned}$$

where $V_{i}^{t,*}$ denotes the stationary solution obtained during the first stage of the reconstruction step in the *i*-th cell at time *t*. Then, the quadrature formula is used to approximate the integral:

63 $$\begin{aligned} \overline{V}_i'(t) = -\frac{1}{\varDelta r} \Bigg ( F_{i+\frac{1}{2}}-F(V_{i}^{t,*}(r_{i+\frac{1}{2}}), r_{i+\frac{1}{2}})+F(V_{i}^{t,*}(r_{i-\frac{1}{2}}),r_{i-\frac{1}{2}}) - F_{i-\frac{1}{2}} \nonumber \\ - \varDelta r \sum _{m=1}^{M} \beta _{m}\big [S(P^t_i(r_{i}^{m}),r_{i}^{m})-S(V^{t,*}(r_{i}^{m}), r_{i}^{m})\big ] \Bigg ). \;\; \end{aligned}$$

Then, it can be easily checked that the right-hand side of (63) vanishes when computed starting from the cell-averages of a stationary solution $V^*$, i.e. the method is well-balanced in the sense of Definition 1.

### First-Order Exactly Fully Well-Balanced Scheme

In the first-order case we use the mid-point rule:

64 $$\begin{aligned} \int _{r_{i-\frac{1}{2}}}^{r_{i+\frac{1}{2}}} f(r) dr \approx \varDelta r f(r_{i}). \end{aligned}$$

The stages for obtaining the reconstruction operator $P_{i}$ are reduced to:

1. Find, if possible, the stationary solution $V_{i}^{*}(r)$ such that:
  65 $$\begin{aligned} V_{i}^{*}(r_{i}) = \overline{V}_{i}^{n}. \end{aligned}$$
  This stationary solution is characterized by
  66 $$\begin{aligned}  &   r^2\frac{\left( 1-(\nu _{i}^{*}(r))^2\right) ^{\frac{1-k^2}{2k^2}}(\nu _{i}^{*}(r)-B(r))}{\left( 1-B(r)\nu _{i}^{*}(r)\right) ^{1/k^2}}=C_{1,i}^{n}, \end{aligned}$$
  67 $$\begin{aligned}  &   r^2\left( \frac{\nu _{i}^{*}(r)-B(r)}{1-(\nu _{i}^{*}(r))^2}\right) \left( 1-B(r)\nu _{i}^{*}(r)\right) \mu _{i}^{*}(r)=C_{2,i}^{n}, \end{aligned}$$
  where
  68 $$\begin{aligned} C_{1,i}^{n} = r_i^2\frac{\left( 1-(\nu _{i}^{n})^2\right) ^{\frac{1-k^2}{2k^2}}(\nu _{i}^{n}-B(r_i))}{\left( 1-B(r_i)\nu _{i}^{n}\right) ^{1/k^2}}, \end{aligned}$$
  69 $$\begin{aligned} C_{2,i}^{n} = r_i^2\left( \frac{\nu _{i}^{n}-B(r_i)}{1-(\nu _{i}^{n})^2}\right) \left( 1-B(r_i)\nu _{i}^{n}\right) \mu _{i}^{n}, \end{aligned}$$
  $$\begin{aligned} B(r) = \sqrt{R/r}. \end{aligned}$$
  Given $r=a$ we are able to compute $V_{i}^{*}(a)$ solving this system. We use a numerical solver for nonlinear equations such as Newton’s method or the bisection method taking into account the study we carried out in Subsection 2.4.
2. We compute the fluctuation:
  $$\begin{aligned} \overline{W}_{i} = \overline{V}_{i} - V_{i}^{*}(r_{i}) = 0. \end{aligned}$$
  This is trivial since we have (65).
3. In this case $Q_{i}(r) \equiv 0$ since it is applied to $\{\overline{W}_{i}=0\}$.
4. Therefore, our reconstruction operator is given exactly fully well-balanced reconstruction operator is given by:
  70 $$\begin{aligned} P_{i}(r) = V_{i}^{*}(r). \end{aligned}$$

After defining the well-balanced reconstruction procedure, the numerical method is expressed by equation (63), where the mid-point rule is once again applied to compute the numerical source term. Note that, in this case, we have:

71 $$\begin{aligned} \overline{V}_i'(t) = -\frac{1}{\varDelta r} \bigg ( F_{i+\frac{1}{2}}-F(V_{i}^{t,*}(r_{i+\frac{1}{2}}), r_{i+\frac{1}{2}})+F(V_{i}^{t,*}(r_{i-\frac{1}{2}}),r_{i-\frac{1}{2}}) - F_{i-\frac{1}{2}}\bigg ), \end{aligned}$$

being $F_{i+\frac{1}{2}} = F(V^{t,-}_{i+\frac{1}{2}}, V^{t,+}_{i+\frac{1}{2}})$, where the reconstructed states are given by:

$$ V^{t,-}_{i+\frac{1}{2}} = P^t_i(r_{i+\frac{1}{2}}) = V_{i}^{*}(r_{i+\frac{1}{2}}), \quad V^{t,+}_{i+\frac{1}{2}} = P^t_{i+1}(r_{i+\frac{1}{2}}) = V_{i+1}^{*}(r_{i+\frac{1}{2}}). $$

Finally, we integrate on time using the Euler’s method and we obtain:

72 $$\begin{aligned} \overline{V}_{i}^{n+1} = \overline{V}_{i}^{n} -\frac{\varDelta t}{\varDelta r} \bigg ( F_{i+\frac{1}{2}}-F(V_{i}^{n,*}(r_{i+\frac{1}{2}}), r_{i+\frac{1}{2}})+F(V_{i}^{n,*}(r_{i-\frac{1}{2}}),r_{i-\frac{1}{2}}) - F_{i-\frac{1}{2}}\bigg ). \end{aligned}$$

### Second-Order Exactly Fully Well-Balanced Scheme

Again the mid-point rule will be use to compute the integrals. The stages for obtaining the reconstruction operator $P_{i}$ are reduced to:

1. This first step is similar to the first-order one. We obtain the stationary solution such as (65).
2. We compute the fluctuations:
  $$\begin{aligned} \overline{W}_{i-1} = \overline{V}_{i-1} - V_{i}^{*}(r_{i-1}), \end{aligned}$$
  $$\begin{aligned} \overline{W}_{i} = \overline{V}_{i} - V_{i}^{*}(r_{i}) = 0, \end{aligned}$$
  $$\begin{aligned} \overline{W}_{i+1} = \overline{V}_{i+1} - V_{i}^{*}(r_{i+1}), \end{aligned}$$
  where the mid-point rule has been used again to compute cell-averages.
3. Apply the minmod reconstruction operator $Q_i$ (see [47]) to $\{\overline{W}_{i-1}, \overline{W}_{i}, \overline{W}_{i+1}\}$:
  73 $$\begin{aligned} Q_{i}(r) = \overline{W}_i + \text {minmod}\left( \frac{\overline{W}_i - \overline{W}_{i-1}}{\varDelta r}, \frac{\overline{W}_{i+1} - \overline{W}_i}{\varDelta r}\right) (r - x_i), \end{aligned}$$
  where
  $$ \text {minmod}(a, b) = {\left\{ \begin{array}{ll} \min (a, b) &  \text {if } a, b > 0, \\ \max (a, b) &  \text {if } a, b < 0, \\ 0 &  \text {otherwise,} \end{array}\right. } $$
  and it is applied variable by variable.
4. Finally we obtain the following reconstruction operator:
  74 $$\begin{aligned} P_{i}(r) = V_{i}^{*}(r) + Q_{i}(r). \end{aligned}$$

After defining the well-balanced reconstruction procedure, the numerical method is expressed by equation (63), where the mid-point rule is once again applied to compute the numerical source term, so:

$$\int _{r_{i-\frac{1}{2}}}^{r_{i+\frac{1}{2}}} \big [S(P^t_i(r),r)-S(V_i^{t,*}(r), r)\big ] \, dr = \varDelta r \, \big [S(P^t_i(r_{i}),r_{i}^{m})-S(V_i^{t,*}(r_{i}), r_{i})\big ] = 0, $$

since $$P^t_i(r_{i}) = V_i^{t,*}(r_{i})$$.

Therefore, in this case the scheme reduces to:

75 $$\begin{aligned} \overline{V}_i'(t) = -\frac{1}{\varDelta r} \bigg ( F_{i+\frac{1}{2}}-F(V_{i}^{t,*}(r_{i+\frac{1}{2}}), r_{i+\frac{1}{2}})+F(V_{i}^{t,*}(r_{i-\frac{1}{2}}),r_{i-\frac{1}{2}}) - F_{i-\frac{1}{2}}\bigg ), \end{aligned}$$

being $F_{i+\frac{1}{2}} = F(V^{t,-}_{i+\frac{1}{2}}, V^{t,+}_{i+\frac{1}{2}})$, where the reconstructed states are given by:

$$ V^{t,-}_{i+\frac{1}{2}} = V_{i}^{*}(r_{i+\frac{1}{2}}) + Q_{i}(r_{i+\frac{1}{2}}), \quad V^{t,+}_{i+\frac{1}{2}} = V_{i+1}^{*}(r_{i+\frac{1}{2}})+ Q_{i+1}(r_{i+\frac{1}{2}}). $$

Finally, the time discretization is carried out using the second-order TVD Runge-Kutta method:

$$\begin{aligned}&\overline{V}_{i}^{(1)} = \overline{V}_{i}^{n} + \varDelta t \, {L_i(\{\overline{V}_{j}^{n}\}; \varDelta x)},\\&\overline{V}_{i}^{n+1} = \frac{1}{2}\overline{V}_{i}^{n} + \frac{1}{2}\overline{V}_{i}^{(1)} + \frac{1}{2}\varDelta t \, {L_i(\{\overline{V}_{j}^{(1)}\}; \varDelta x)}, \end{aligned}$$

where ${L_i(\{\overline{V}_{j}\}; \varDelta x)}$ represents the right-hand side of (75) (see [17]).

### Limited Well-Balanced Reconstruction Operator for Preserving Only Global Stationary Solutions in a Smooth Way

**Fig. 10:**
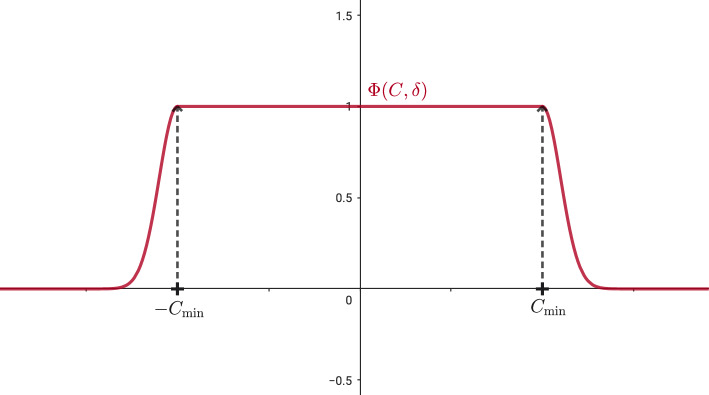
Graphic of function $\varPhi (C,\delta )$ (76)

The previous reconstruction operator is able to preserve all stationary solutions but the only ones with physical sense are those defined globally, i.e., in the $(0, \infty )$ interval. Due to this fact, we need to limit our reconstruction operator and in order to avoid oscillation we need to do it in a smooth way. Based on a similar strategy in [16], we introduce the following smooth limitation to our reconstruction operator:

1. We define the limiting function:
  76 $$\begin{aligned} \varPhi (C,\delta ) = {\left\{ \begin{array}{ll} e^{-\frac{(C+C_{\text {min}})^2}{2\delta ^2}}, &  \text {if } C<-C_{\text {min}} \\ \ \\ 1, &  \text {if } C\in [-C_{\text {min}},C_{\text {min}}], \\ \\ e^{-\frac{(C-C_{\text {min}})^2}{2\delta ^2}}, &  \text {if } C\in C>C_{\text {min}}, \end{array}\right. } \end{aligned}$$
  where $C_{min} = \varPsi (B_{\text {min}})$ is given in (47). See Fig. 10.
2. The reconstruction operator will be given by:
  77 $$\begin{aligned} P_{i}(r) = \varPhi (C_{1,i}^{n}, \delta _{i}^{n})V_{i}^{*}(r) + \hat{Q}_{i}(r), \end{aligned}$$
  where $V_{i}^{*}$ is the stationary solution that verifies (60),
  $$ \hat{Q}_{i}(r) = \hat{Q}_{i}(r; \{\hat{W}_{j}\}_{j\in S_{i}}), $$
  is again a standard reconstruction operator but applied to the fluctuations given by
  78 $$\begin{aligned} \hat{W}_{j} = V_{j} - \varPhi (C_{1,i}^{n}, \delta _{i}^{n})\sum _{m=1}^{M} \beta _{m} V_{i}^{*}(r_{j}^{m}), \ \ j\in S_{i}. \end{aligned}$$

#### Remark 2

$C_{1,i}^{n}$ is given by (68) and $\delta _{i}^{n}=0.1$.

#### Remark 3

We observe that this limitation of the reconstruction operators affects only the first- and second-order schemes in the reconstructed states but not in the computation of the source terms since $\hat{Q}_{i}(r_i)=V_{i}^{*}(r_i)- \varPhi (C_{1,i}^{n}, \delta _{i}^{n})V_{i}^{*}(r_i)$. Indeed

$$\begin{aligned} P_{i}(r_{i}) = \varPhi (C_{1,i}^{n}, \delta _{i}^{n})V_{i}^{*}(r_i) + V_{i}^{*}(r_i) - \varPhi (C_{1,i}^{n}, \delta _{i}^{n})V_{i}^{*}(r_i) = V_{i}^{*}(r_i). \end{aligned}$$

#### Remark 4

The exactly fully well-balanced property in Definition 1 is kept for global stationary solutions since the reconstruction operator is reduced to the exactly fully well-balanced one in these cases.

### Numerical Flux

What remains to be defined are the numerical fluxes $F_{i+\frac{1}{2}}$ that we use in the scheme. In our case, we are going to use an HLL-like method written in PVM form (see [10]) that reads as:

79 $$\begin{aligned} F_{i+\frac{1}{2}} = F(V_l, V_r) = \frac{1}{2}\Big (F(V_r, r_r) +F(V_r,r_r) - \alpha _0(V_r-V_l) - \alpha _1(F(V_r, r_r)-F(V_l,r_l))\Big ), \end{aligned}$$

where

$$\begin{aligned} \alpha _0 = \frac{S_r|S_l|-S_l|S_r|}{S_r-S_l}, \ \ \alpha _1 = \frac{|S_r|-|S_l|}{S_r-S_l}, \end{aligned}$$

being

$$\begin{aligned} S_l = \min (\lambda _1(V_l,r_l), \lambda _1(V_r,r_r)), \ \ \max (\lambda _2(V_l,r_l), \lambda _2(V_r,r_r)). \end{aligned}$$

In the case $p = k^2\mu$ we have

$$\begin{aligned} \lambda _1(V,r) = \frac{\nu -k}{1-k\,\nu }-B(r), \ \ \lambda _2(V,r) = \frac{\nu +k}{1+k\,\nu }-B(r). \end{aligned}$$

## Numerical Tests

In this section we consider the model with $k= 0.3$ in the radial domain [0.5, 10]. The event horizon is located at $r=R=1$. Unless otherwise stated, the number of cells is set to $N=1000$, so $\varDelta r = (10-0.5)/1000 = 0.0095$. The time step is given by the usual CFL condition

$$\begin{aligned} \varDelta t = CFL\, \frac{\varDelta r}{\lambda _{\text {max}}^n}, \end{aligned}$$

where $\lambda _{\text {max}}^n$ is the maximum of all eigenvalues in absolute value evaluated at time *n* in all cells and $CFL \in (0,1)$. Here, we take $CFL = 0.5$. The following labels will be used for the different methods considered in the numerical experiments:

- O1_noWB: First-order standard (non well-balanced) HLL method.
- O2_noWB: Second-order standard (non well-balanced) HLL method.
- O1_WB: First-order exactly fully well-balanced HLL method with modified reconstruction operator given in Subsection 3.5.
- O2_WB: Second-order exactly fully well-balanced HLL method with modified reconstruction operator given in Subsection 3.5.

### Boundary Conditions

To define the boundary conditions for the different test cases, we must note that the left boundary of the domain lies inside the event horizon. As can be seen in Fig. 1a looking at the sonic point graphs, in this region both the eigenvalues $\lambda _1$ and $\lambda _2$ are negative, indicating that all the waves propagate toward the black hole. Consequently, it is not physically meaningful to impose a boundary condition on the left side of the domain. Therefore, boundary conditions will only be imposed on the right side when necessary.

### Well-Balanced Tests

The following tests are devoted to show how the numerical methods preserve the global stationary solutions of the model (see Section 2.4.1). For the chosen value of *k*, one has $C_{min} = 12.49998$.

#### Test 1: Global Supersonic Stationary Solution with Negative $C_1$

In this first test we consider a stationary solution of family 1 (see Section 2.4.1). More precisely, we consider the following initial condition for the variables $\mu$ and $\nu$:

80 $$\begin{aligned} W_{0}(r) = W_{0}^{*}(r) = (\mu ^{*}_{0}(r), \nu _{0}^{*}(r))^T, \end{aligned}$$

where $W_{0}^{*}(r)$ is the global supersonic stationary solution defined by the constants

81 $$\begin{aligned} C_{1} = -0.00169888, \ \ C_{2} = -822.301 \end{aligned}$$

in (41). The boundary condition on the right is fixed as

$$\begin{aligned} W_{0}(10)=(\mu _{0}(10), \nu _{0}(10))^T=(1, -0.9)^T. \end{aligned}$$

**Fig. 11:**
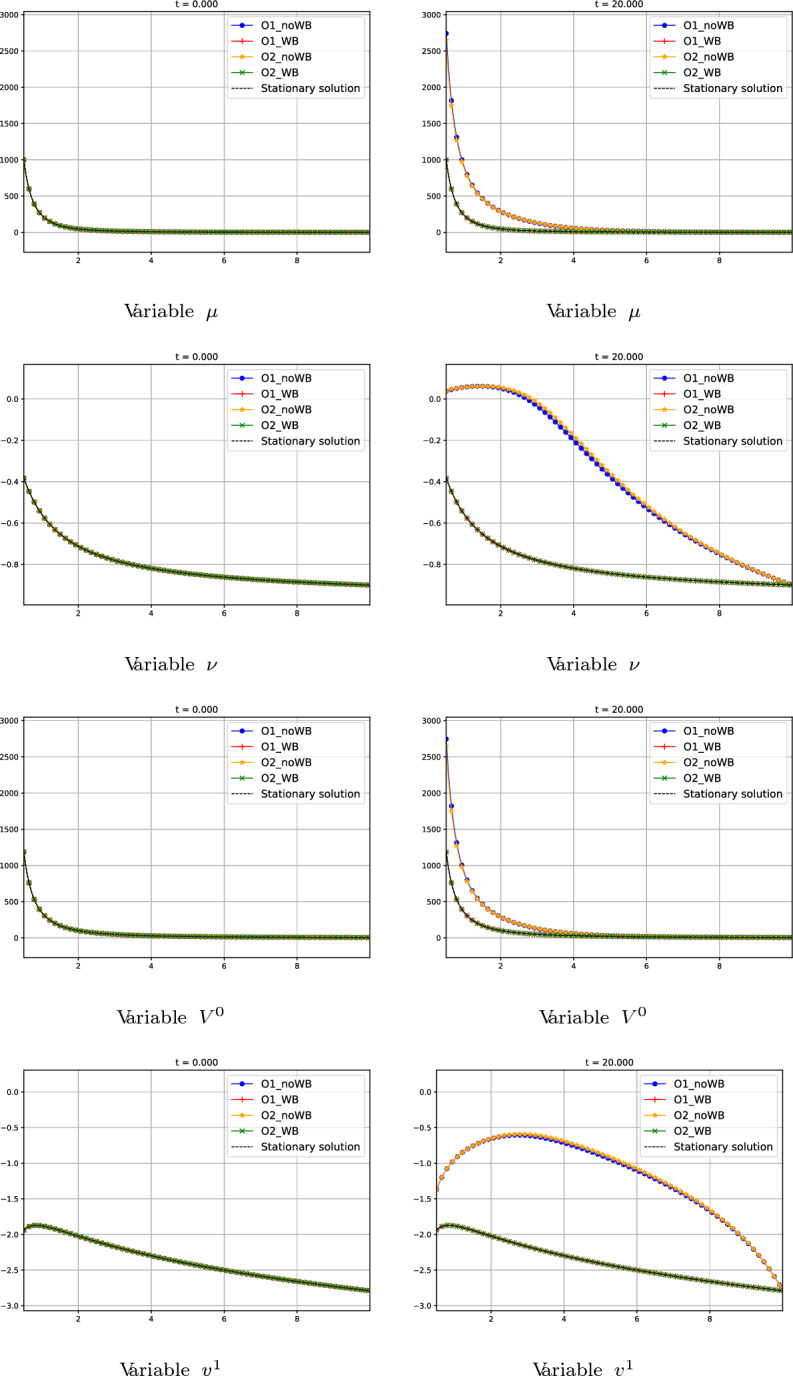
Test 1: numerical solution of first-, second-order well-balanced and non well-balanced methods at different times. First line: variable $\mu$. Second line: variable $\nu$. Third line: variable $V^0$. Fourth line: variable $v^1$. First column: initial data at $t=0$. Second column: values at $t=5$. Third column: values at $t=20$. Blue curves correspond to first-order non well-balanced method. Red curves correspond to first-order well-balanced method. Yellow curves correspond to second-order non well-balanced method. Green curves correspond to second-order well-balanced method. Black curves correspond to the initial stationary solution

In Fig. 11 we show the numerical results given by the different methods: it can be observed that only the well-balanced schemes are capable of preserving the stationary solution. The numerical solutions given by the non well-balanced ones converge as time goes by to a limit that is not a stationary solution of the PDE system. In Table 1$L^{1}$ errors between the initial stationary solution and the numerical solution at time $t = 20$ are shown to prove the well-balanced property of the methods.

**Table 1:** Test 1: $L^{1}$ errors at time $t = 20$

Scheme	$||\varDelta \mu ||_1$	$||\varDelta \nu ||_1$	$||\varDelta V^0||_1$	$||\varDelta v^1||_1$
O1_WB	9.44E-11	3.80E-13	1.11E-10	3.24E-12
O2_WB	2.70E-10	6.27E-13	2.26E-10	4.07E-12
O1_noWB	1225.85	3.95	986.36	11.24
O2_noWB	1198.53	4.03	957.92	11.37

#### Test 2: Global Supersonic Stationary Solution with Positive $C_1$

In this test we consider as initial condition the global supersonic stationary solution

82 $$\begin{aligned} W_{0}(r) = W_{0}^{*}(r) = (\mu ^{*}_{0}(r), \nu _{0}^{*}(r))^T, \end{aligned}$$

defined by the constants

83 $$\begin{aligned} C_{1} = 0.544627, \ \ C_{2}=219.804 \end{aligned}$$

in (41). The boundary condition on the right is fixed as

$$\begin{aligned} W_{0}(10)=(\mu _{0}(10), \nu _{0}(10))^T=(1, 0.9)^T. \end{aligned}$$

According to Theorem 1, $V^0$ is not bounded close to the event horizon, so this stationary solution does not belong to any of the families listed in Section 2.4.1. The unbounded character of $\mu$ makes this numerical test particularly challenging.

**Fig. 12:**
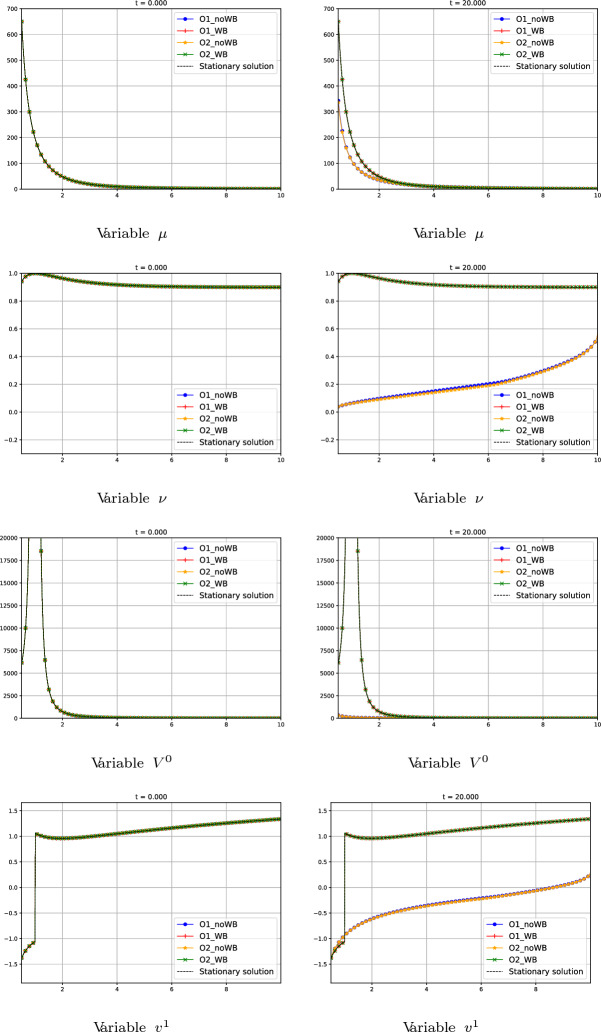
Test 2: numerical solution of first-, second-order well-balanced and non well-balanced methods at different times. First line: variable $\mu$. Second line: variable $\nu$. Third line: variable $V^0$. Fourth line: variable $v^1$. First column: initial data at $t=0$. Second column: values at $t=1$. Third column: values at $t=20$. Blue curves correspond to first-order non well-balanced method. Red curves correspond to first-order well-balanced method. Yellow curves correspond to second-order non well-balanced method. Green curves correspond to second-order well-balanced method. Black curves correspond to the initial stationary solution

In Fig. 12 we show the numerical results given by the different methods. Again, while the well-balanced methods preserve the initial condition the non well-balanced ones converge in time to a limit which is not a stationary solution.

#### Test 3: Order Test

Let us consider now the following initial condition:

84 $$\begin{aligned} W_{0}(r) = W_{0}^{*}(r) + \delta (r), \end{aligned}$$

where $W_{0}^{*}(r)$ is again the global supersonic stationary solution of Test 1 defined by the constants

85 $$\begin{aligned} C_{1} = -0.00169888, \ \ C_{2}=-822.301 \end{aligned}$$

in (41) and $\delta (r)=(- 0.01e^{-200(r-6)^2}, 0 )^T$. The boundary condition on the right is fixed as

$$\begin{aligned} W_{0}(10)=(\mu _{0}(10), \nu _{0}(10))^T=(1, -0.9)^T. \end{aligned}$$

The numerical experiment is run until time $t = 0.5$ in which the solution is smooth. A reference solution has been computed using 3200 mesh points. In Table 2 we show the numerical errors in $L^{1}$ norm for the first- and second-order exactly fully well-balanced schemes. The expected order of accuracy is achieved in both cases.

**Table 2:** Test 3: order of accuracy for the first- and second-order exactly fully well-balanced scheme: $L^{1}$ errors $||\varDelta \cdot ||_1$ at time $t=0.5$

Number of cells	$||\varDelta \mu ||_1$	Order	$||\varDelta \nu ||_1$	Order	$||\varDelta V^0||_1$	Order	$||\varDelta v^1||_1$	Order
First-order								
100	1.57e+00	0.00	1.39e-03	0.00	1.79e+00	0.00	1.19e-02	0.00
200	4.24e-01	1.89	9.88e-04	0.49	5.87e-01	1.61	9.20e-03	0.38
400	1.27e-01	1.74	6.30e-04	0.65	2.40e-01	1.29	6.04e-03	0.61
800	4.36e-02	1.55	3.42e-04	0.88	1.14e-01	1.07	3.31e-03	0.87
1600	1.39e-02	1.65	1.33e-04	1.36	4.47e-02	1.35	1.30e-03	1.35
Second-order								
100	1.58e+00	0.00	1.38e-03	0.00	1.80e+00	0.00	1.19e-02	0.00
200	4.26e-01	1.89	7.87e-04	0.82	6.03e-01	1.58	7.27e-03	0.72
400	1.27e-01	1.74	3.80e-04	1.05	2.31e-01	1.39	3.62e-03	1.00
800	4.00e-02	1.67	1.63e-04	1.23	9.22e-02	1.32	1.57e-03	1.21
1600	9.85e-03	2.02	4.21e-05	1.95	2.61e-02	1.82	4.10e-04	1.94

#### Test 4: Perturbation of a Global Supersonic Stationary Solution

We now consider the following initial condition:

86 $$\begin{aligned} W_{0}(r) = W_{0}^{*}(r) + \delta (r), \end{aligned}$$

where $W_{0}^{*}(r)$ is again the global supersonic stationary solution of Test 1 defined by the constants

87 $$\begin{aligned} C_{1} = -0.00169888, \ \ C_{2}=-822.301 \end{aligned}$$

in (41) and $\delta (r)$ is in this case a larger perturbation given by

$$\begin{aligned} \delta (r)=(-0.1 \, e^{-200(r-6)^2}, 0 )^T. \end{aligned}$$

The boundary condition on the right is fixed again as

$$\begin{aligned} W_{0}(10)=(\mu _{0}(10), \nu _{0}(10))^T=(1, -0.9)^T. \end{aligned}$$

**Fig. 13:**
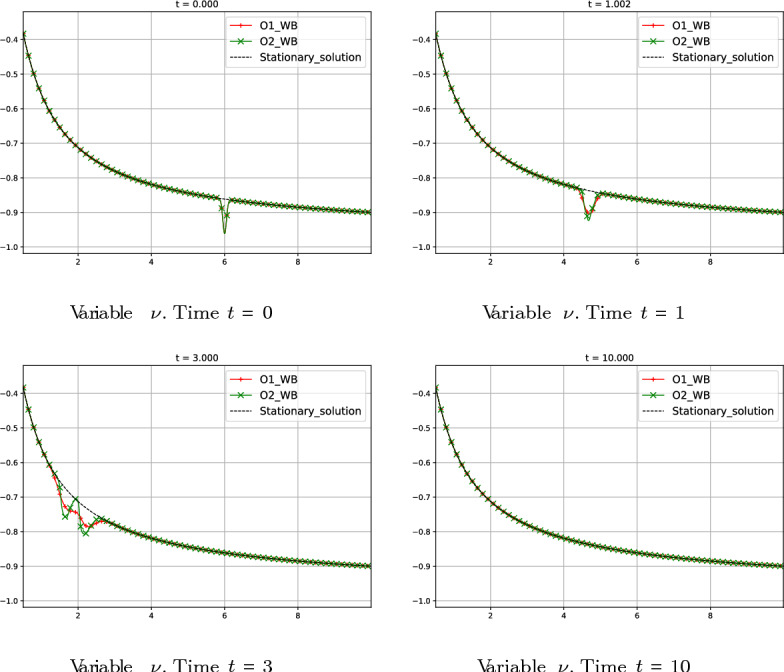
Test 4: numerical solution of first- and second-order well-balanced methods at different times. Red curves correspond to first-order well-balanced method. Green curves correspond to second-order well-balanced method. Black curves correspond to the stationary solution without perturbation

The numerical experiment is run until the final time $t = 10$ to follow the evolution of the waves generated by the initial perturbation. In Fig. 13 we show the numerical solution given by the first- and second-order well-balanced schemes and the undisturbed stationary solution. We observe that, in both cases, the stationary solution is recovered once the waves leave the domain. Observe that, since the regime is supersonic and the two eigenvalues are negative, the waves travel to the left.

#### Test 5: Convergence in Time to a Global Stationary Solution with $C_1 = -C_{\text {min}}$

Let us consider the following initial condition:

88 $$\begin{aligned} W_{0}(r) = (1, 0.4\sin (r)-0.5)^T, \end{aligned}$$

together with the boundary condition

$$\begin{aligned} W^*_{0}(10)=(\mu ^*_{0}(10), \nu ^*_{0}(10))^T= (1, -0.3219175)^T, \end{aligned}$$

where $W_{0}^{*}(r)$ is the global stationary solution of family 7 (see Section 2.4.1) defined by the constants

89 $$\begin{aligned} C_{1} = -C_{\text {min}} = -12.49998, \ \ C_{2}=-78.43955 \end{aligned}$$

in (41). This solution is supersonic everywhere but in $\tilde{R}$ where it is sonic. It is continuous but not differentiable at $\tilde{R}$.

**Fig. 14:**
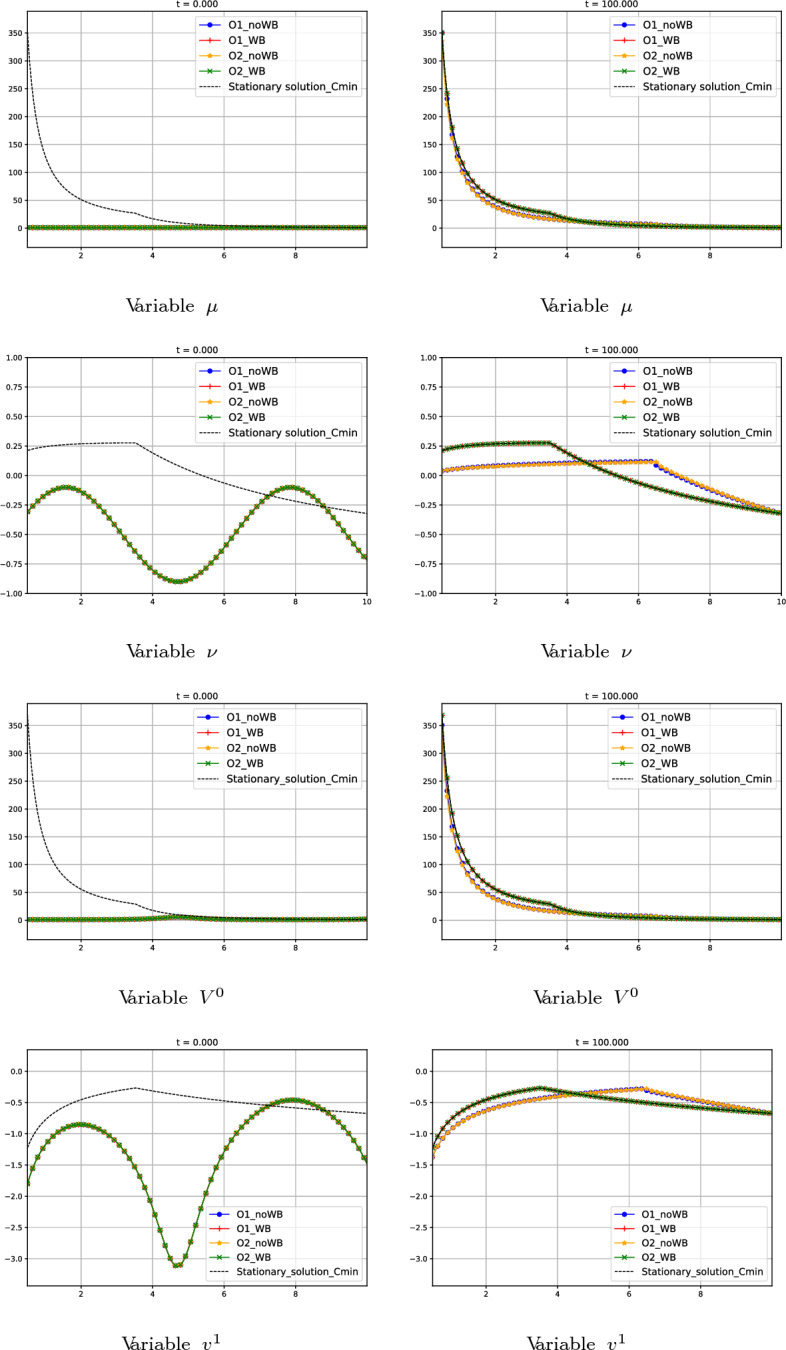
Test 5: numerical solution of first-, second-order well-balanced and non well-balanced methods at different times. First line: variable $\mu$. Second line: variable $\nu$. Third line: variable $V^0$. Fourth line: variable $v^1$. First column: initial data at $t=0$. Second column: values at $t=5$. Third column: values at $t=100$. Blue curves correspond to first-order non well-balanced method. Red curves correspond to first-order well-balanced method. Yellow curves correspond to second-order non well-balanced method. Green curves correspond to second-order well-balanced method. Black curves correspond to the supersonic stationary solution with $C_1 = -C_{min}$

This test is devoted to check whether or not the different numerical schemes are able to converge in time to the stationary solution determined by the boundary condition starting from an initial condition which is far of it. Fig. 14 shows the numerical results given by the different numerical methods. We observe once again that the numerical solutions provided by the well-balanced schemes converge in time to the correct stationary solution, while the non well-balanced ones seem to converge to a limit which is not a stationary solution of the system.

#### Test 6: Comparison Between Limited and Standard Well-Balanced Reconstruction Operators

Let us consider the following initial condition:

90 $$\begin{aligned} W_{0}(r) = (1, 0.2\sin (r))^T. \end{aligned}$$

The boundary condition on the right is fixed as

$$\begin{aligned} W^*_{0}(10)=(\mu ^*_{0}(10), \nu ^*_{0}(10))^T = (1, -0.3219175)^T, \end{aligned}$$

where $W_{0}^{*}(r)$ is again the global supersonic stationary solution of Test 5, defined by the constants

91 $$\begin{aligned} C_{1} = -C_{\text {min}}= -12.49998, \ \ C_{2}=-78.43955 \end{aligned}$$

in (41).

**Fig. 15:**
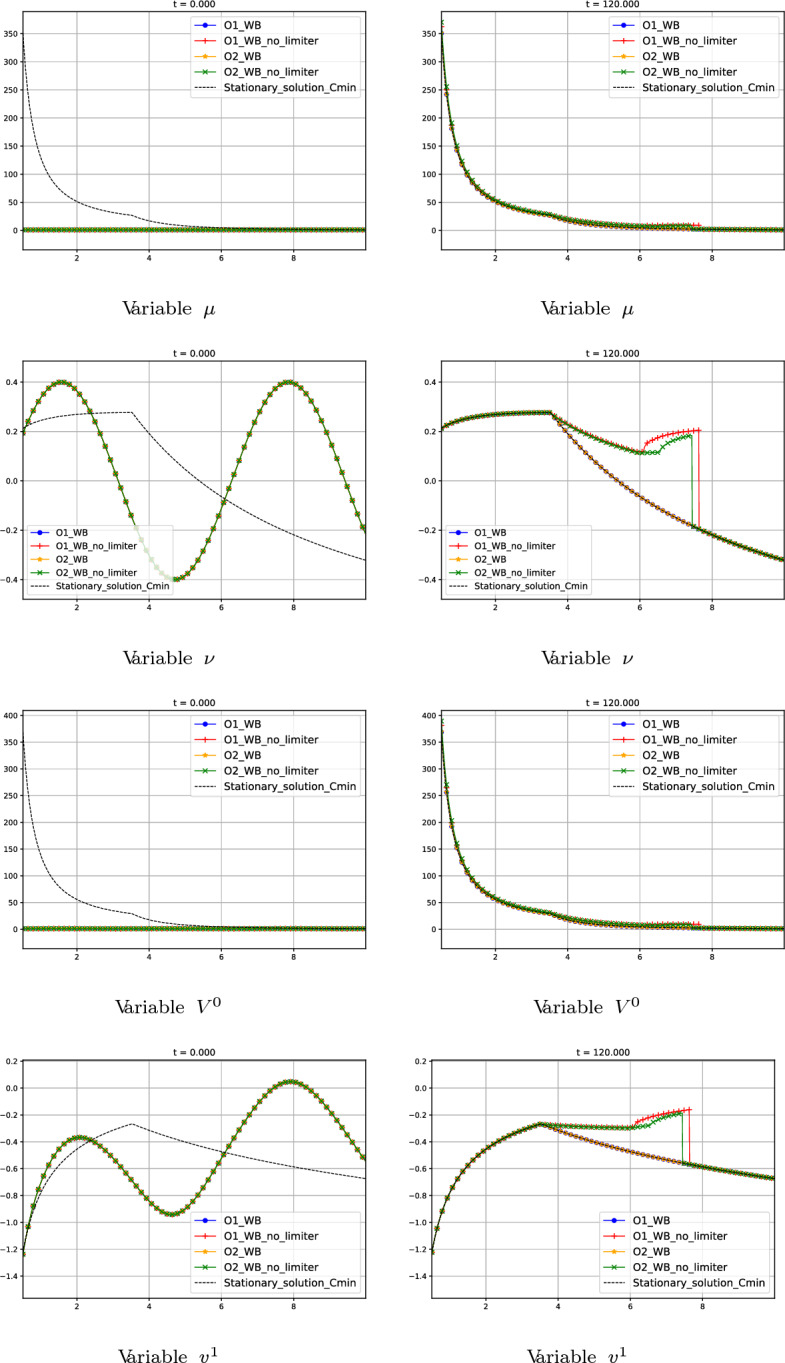
Test 6: numerical solution of first- and second-order well-balanced methods with and without limitation described in Subsection 3.5 at different times. First line: variable $\mu$. Second line: variable $\nu$. Third line: variable $V^0$. Fourth line: variable $v^1$. First column: initial data at $t=0$. Second column: values at $t=5$. Third column: values at $t=120$. Blue curves correspond to first-order well-balanced method with limitation. Red curves correspond to first-order well-balanced method without limitation. Yellow curves correspond to second-order well-balanced method with limitation. Green curves correspond to second-order well-balanced method without limitation. Black curves correspond to the supersonic stationary solution with $C_1 = -C_{min}$

This test is devoted to showing the advantages of using the limited well-balanced reconstruction operator introduced in Section 3.5 instead of the standard well-balanced reconstruction operator. In Fig. 15 we show the numerical results given by the first- and second-order well-balanced methods with and without this limitation. We observe that the schemes without limitation converge to a different stationary solution that involves a transonic stationary shock wave, while the one with limitation is able to converge to the smooth stationary solution determined by the boundary condition.

#### Test 7: Smooth Transonic Stationary Solution with $C_1=-C_{min}$

We now consider the following initial condition:

92 $$\begin{aligned} W_{0}(r) = W_{0}^{*}(r) = (\mu ^{*}_{0}(r), \nu _{0}^{*}(r))^T, \end{aligned}$$

where $W_{0}^{*}(r)$ is the smooth transonic stationary solution of family 2 (see Section 2.4.1) defined by the constants.

93 $$\begin{aligned} C_{1} = -C_{\text {min}} = -12.49998, \ \ C_{2}=-78.43955 \end{aligned}$$

in (41). The boundary condition on the right is fixed now as

$$\begin{aligned} W^*_{0}(10)=(\mu ^*_{0}(10), \nu ^*_{0}(10))^T = (1, 0.2465837)^T. \end{aligned}$$

**Fig. 16:**
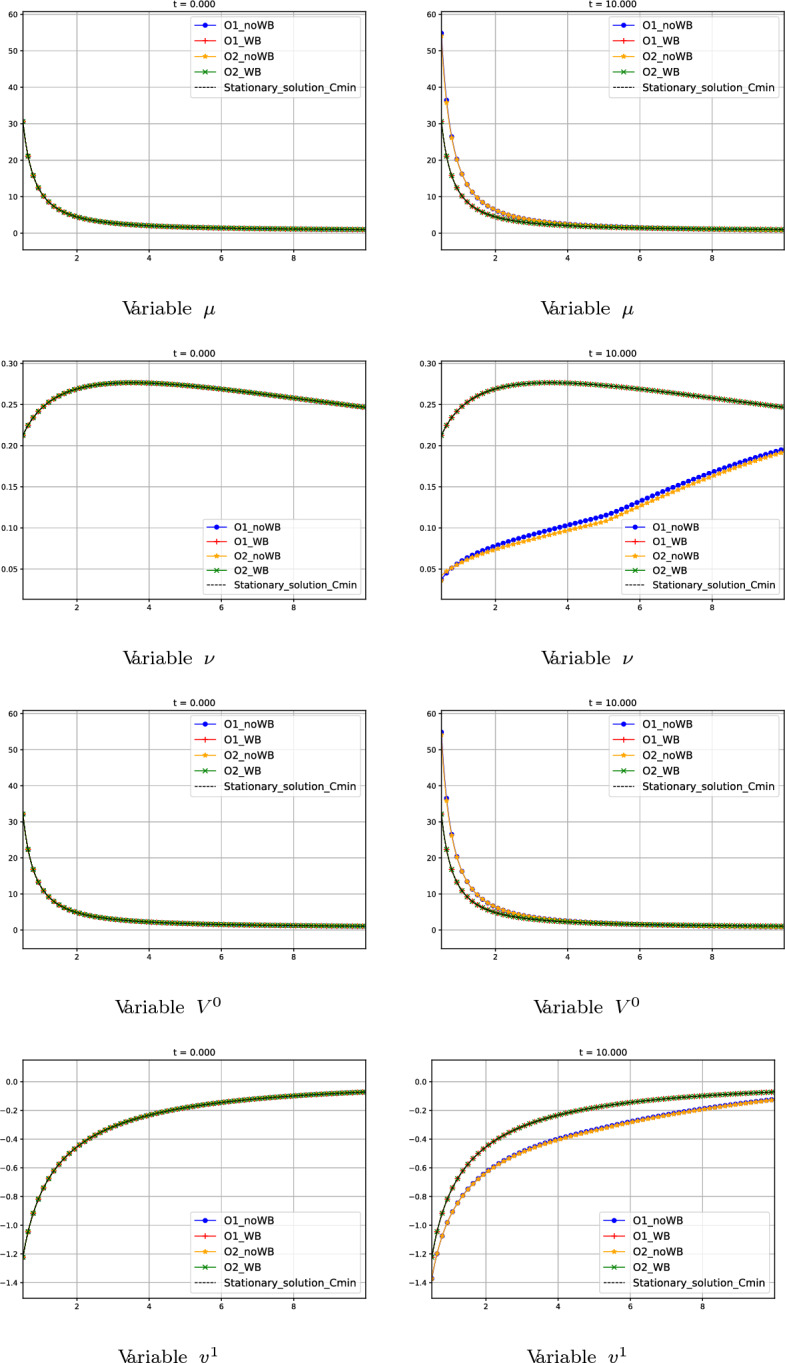
Test 7: numerical solution of first-, second-order well-balanced and non well-balanced methods at different times. First line: variable $\mu$. Second line: variable $\nu$. Third line: variable $V^0$. Fourth line: variable $v^1$. First column: initial data at $t=0$. Second column: values at $t=1$. Third column: values at $t=10$. Blue curves correspond to first-order non well-balanced method. Red curves correspond to first-order well-balanced method. Yellow curves correspond to second-order non well-balanced method. Green curves correspond to second-order well-balanced method. Black curves correspond to the initial smooth transonic stationary solution with $C_1 = -C_{min}$

In Fig. 16 the numerical results given by the different methods are shown. As in previous tests, the non well-balanced schemes are not able to preserve the stationary solution while the well-balanced ones keep it. In Table 3$L^{1}$ errors between the initial stationary solution and the numerical solution at time $t = 10$ are shown to check the well-balanced property of the methods.

**Fig. 17:**
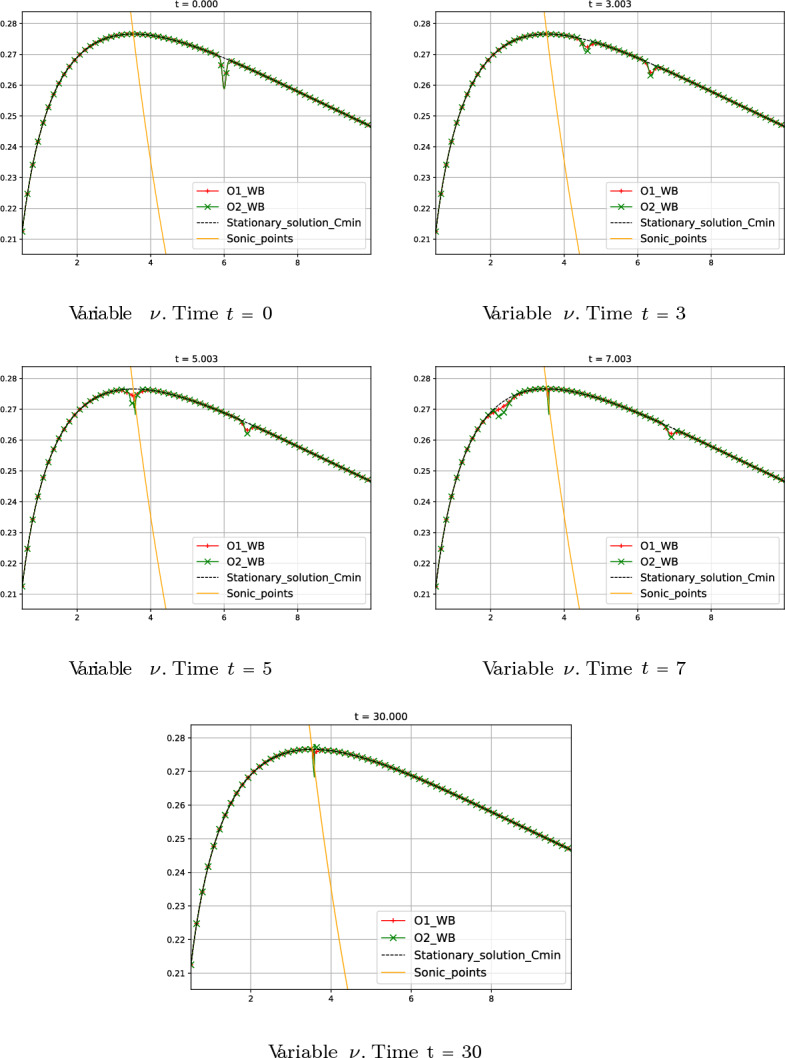
Test 8: numerical solution of first- and second-order well-balanced methods at different times. Red curves correspond to first-order well-balanced method. Green curves correspond to second-order well-balanced method. Black curves correspond to the smooth transonic stationary solution without perturbation. Orange line corresponds to the sonic points in the domain

**Table 3:** Test 7: $L^{1}$ errors at time $t = 10$

Scheme	$||\varDelta \mu ||_1$	$||\varDelta \nu ||_1$	$||\varDelta V^0||_1$	$||\varDelta v^1||_1$
O1_WB	8.29E-13	4.32E-14	9.23E-13	4.09E-14
O2_WB	9.00E-13	3.86E-14	1.00E-12	3.67E-14
O1_noWB	12.76	1.33	11.19	1.26
O2_noWB	12.66	1.37	11.08	1.31

#### Test 8: Perturbation of a Smooth Transonic Stationary Solution with $C_1 = -C_{min}$

In this test, we consider the initial condition

94 $$\begin{aligned} W_{0}(r) = W_{0}^{*}(r) + \delta (r), \end{aligned}$$

where $W_{0}^{*}(r)$ is again the smooth transonic stationary solution considered in the previous test and $\delta (r)$ is in this case an initial perturbation given by $\delta (r)=(-0.01 \, e^{-200(r-6)^2}, 0 )^T$. The boundary condition on the right is as in the previous test. Fig. 17 shows the numerical solution given by the first- and second-order well-balanced schemes and the undisturbed stationary solution. Observe that since the initial perturbation is located in the subsonic regime region, the generated waves travel in both directions. Both waves going to the right and to the left travel to the boundaries, but the latter leaves a perturbation behind it as it passes by the sonic point. This perturbation only affects two points and decreases as the mesh is refined, so it seems to be a numerical artifact. This is a very challenging numerical test and further refinement will be necessary to avoid this artifact.

## Final Remarks

We studied the problem of a perfect fluid moving in a fixed Schwarzschild spacetime. To do so we have considered the general relativistic Euler Equation in the Gullstrand-Painlevé coordinates. The resulting system allows the fluid to cross the event horizon $r=R$ with different negative values for the radial component of the velocity. Then, using these coordinates we do not need to restrict our spatial domain to the region $r>R$ as in Schwarzschild coordinates, the ones used in [26] to address the same problem. Gullstrand-Painlevé coordinates do not have any coordinate singularity but have the drawback of making computations trickier. We studied the hyperbolic behaviour of the system and we obtained its stationary solutions. The implicit expression of these stationary solutions allows us to develop fully well-balanced schemes, i.e., numerical methods that are able to preserve all its stationary solutions. This was the spirit of the work [25], but the main advantage here is that we can consider the whole domain $0< r < \infty$. These schemes are also able to capture their behavior in the face of perturbations with a level of precision that surpasses other numerical schemes. Our investigation has encompassed the exploration of all conceivable types of stationary solutions and the derivation of fully well-balanced methods tailored for the Schwarzschild-Euler Equation in Gullstrand- Painlevé coordinates.

This work has a lot of potential applications in astrophysical scenarios dealing with compact objects. We have shown their effectiveness in the presence of the most extreme compact objects known in the framework of the theory of GR, BHs, in the case of spherical symmetry. First, we can think about a much more precise and efficient computation of modes in equilibrium configurations of compact objects like neutron stars [15]. Second, stability of equilibrium configurations of compact objects in a general way can be explored with more accuracy, for example for the case of spinning scalar boson and Proca stars [20] reviewing previous stability analysis [39]. Finally, a more ambitious astrophysical application, that goes beyond spherical symmetry, is the analysis of the stability of accretion discs around black holes.

## Data Availability

Data are available from the authors upon reasonable request.

## Appendix A Analysis of the Stationary Solutions

### Theorem 1

Suppose that $W(r) = [\mu (r),\nu (r)]^T$ is a smooth stationary solution defined in a neighborhood of $r = R$ that satisfies (41) with $C_1 > 0$. Then:

1. $$\displaystyle \lim _{r \rightarrow R} \nu (r) = 1$$.
2. $$\displaystyle \lim _{r \rightarrow R} \mu (r) = \frac{C_{2} C_{1}^{\frac{2k^2}{1-k^2}}}{R^{2\frac{1+k^2}{1-k^2}}}.$$
3. $V^0(r)$ remains bounded only if $C_2 = 0$, i.e. $\mu \equiv 0$.
4. *W*(*r*) is supersonic for $r \not = R$.

### Proof

1. Let us first compute the limits of $\nu$ as $r \rightarrow R^\pm$. If the stationary solution satisfies (41) with $C_1 > 0$, then:
  - For $r > R$, i.e. for $B < 1$ necessarily $\nu (r) = \nu _{sub}$ or $\nu _{sup}$ with
    $$\begin{aligned} \nu _{sub}\in \left[ B, \frac{k+B}{kB+1}\right) , \ \ \nu _{sup}\in \left( \frac{k+B}{kB+1},1\right) . \end{aligned}$$
    Since $r \rightarrow R^+$ implies $B \rightarrow 1^-$, we obtain
    $$\begin{aligned} \lim _{r \rightarrow R^+} \nu (r) = 1. \end{aligned}$$
  - For $r < R$, i.e. for $B>1$ necessarily $\nu _r = \nu _{sup}$ with
    $$\begin{aligned} \nu _{sup}\in \left( \frac{1}{B}, 1 \right) . \end{aligned}$$
    Since $r \rightarrow R^-$ implies $B \rightarrow 1^+$, we obtain again
    $$\begin{aligned} \lim _{r \rightarrow R^-} \nu (r) = 1. \end{aligned}$$
    Therefore
    $$\begin{aligned} \lim _{r \rightarrow R} \nu (r) = 1. \end{aligned}$$
2. Since the stationary solution is assumed to be continuous and (41) is satisfied for all $r \not = R$, then necessarily
  $$ \lim _{B \rightarrow 1} r^2\frac{|\nu -B|\left( 1-\nu ^2\right) ^{\frac{1-k^2}{2k^2}}}{|1-B\nu |^{1/k^2}}=|C_1| = C_1, $$
  so that
  95 $$\begin{aligned} \lim _{B \rightarrow 1} r^{\frac{4k^2}{1-k^2}}\frac{|\nu -B|^{\frac{2k^2}{1-k^2}}}{|1-B\nu |^{\frac{2}{1-k^2}}}\left( 1-\nu ^2\right) =C_1^{\frac{2k^2}{1-k^2}}. \end{aligned}$$
  We use now the second equation of (41) to obtain $\mu$ by taking the limit as $B \rightarrow 1$ (since $\mu >0$, we can take the absolute value again):
  $$\begin{aligned} \lim _{B\rightarrow 1} \mu  &   = \lim _{B \rightarrow 1} \frac{C_{2} (1-\nu ^{2})}{r^2 |\nu -B| |1-B\nu |} \\  &   = \lim _{B \rightarrow 1} \frac{C_{2} (1-\nu ^{2})}{r^2 |\nu -B| |1-B\nu |} \frac{r^{\frac{4k^2}{1-k^2}}|\nu -B|^{\frac{2k^2}{1-k^2}}}{|1-B\nu |^{\frac{2}{1-k^2}}} \frac{|1-B\nu |^{\frac{2}{1-k^2}}}{r^{\frac{4k^2}{1-k^2}}|\nu -B|^{\frac{2k^2}{1-k^2}}} \\  &   \underset{(95)}{=}\ C_{2} C_{1}^{\frac{2k^2}{1-k^2}} \lim _{B\rightarrow 1} \frac{|1-B\nu |^{\frac{2}{1-k^2}-1}}{r^{2+\frac{4k^2}{1-k^2}}|\nu -B|^{1+\frac{2k^2}{1-k^2}}} \\  &   = C_{2} C_{1}^{\frac{2k^2}{1-k^2}} \lim _{B\rightarrow 1} \frac{|1-B\nu |^{\frac{1+k^2}{1-k^2}}}{r^{2\frac{1+k^2}{1-k^2}}|\nu -B|^{\frac{1+k^2}{1-k^2}}} = \\  &   = C_{2} C_{1}^{\frac{2k^2}{1-k^2}} \lim _{B\rightarrow 1} \left( \frac{|1-B\nu |}{r^{2}|\nu -B|}\right) ^{\frac{1+k^2}{1-k^2}} \\  &   \underset{\left( \frac{1}{r^2} = \frac{B^{4}}{R^{2}}\right) }{=} \frac{C_{2} C_{1}^{\frac{2k^2}{1-k^2}}}{R^{2\frac{1+k^2}{1-k^2}}}\lim _{B\rightarrow 1} \left( B^4 \frac{|1-B\nu |}{|\nu - B|}\right) ^{\frac{1+k^2}{1-k^2}} = \frac{C_{2} C_{1}^{\frac{2k^2}{1-k^2}}}{R^{2\frac{1+k^2}{1-k^2}}}, \end{aligned}$$
  where we have used that
  $$\begin{aligned} \lim _{B\rightarrow 1} \left( \frac{1-B\nu }{\nu - B}\right) = 1. \end{aligned}$$
  In effect, we have that $\nu \equiv \nu (B)$ and $\lim _{B\rightarrow 1} \nu (B) = 1$, so that the previous limit is a 0/0 indetermination. Using L’Hôpital rule, one has
  $$\begin{aligned} \lim _{B\rightarrow 1} \left( \frac{1-B\nu (B)}{\nu (B) - B}\right) = \lim _{B \rightarrow 1} \frac{-\nu (B) - B\nu '(B)}{\nu '(B)-1} = \frac{-1-\nu '(1)}{\nu '(1)-1}, \end{aligned}$$
  where $\nu '(B)$ represents the derivative of $\nu$ with respect to *B*. On the other hand, since $\nu (B) < 1$ for all $B \not = 1$ and $\nu (B) = 1$, the function $\nu$ reaches the maximum at $B = 1$ so that $\nu '(1) = 0.$
3. Taking into account point 2 and (18b), if $$V^0 $$ is bounded one has
  $$\begin{aligned} 0 = \lim _{r \rightarrow R} (1 - \nu ^2)V^0 = \lim _{r \rightarrow R} (1 + k^2 \nu ^2) \mu = (1 + k^2) \frac{C_{2} C_{1}^{\frac{2k^2}{1-k^2}}}{R^{2\frac{1+k^2}{1-k^2}}} . \end{aligned}$$
  Clearly, this is only possible for $C_2 = 0.$
4. According to Case 1, the subsonic stationary solutions satisfy
  $$ \varPsi _+(r)< \nu (r) < \varPsi _-(r),$$
  for $$r \ge R$$, where
  $$\begin{aligned} \varPsi _\pm (r ) = \frac{\sqrt{R} \mp k \sqrt{r}}{\sqrt{r} \mp k \sqrt{R}}. \end{aligned}$$
  $\varPsi _+(r)$ (resp. $\varPsi _-(r)$) gives the value of $\nu$ corresponding to the only possible sonic state at *r* such that $\lambda _+ = 0$ (resp. $\lambda _- = 0$). Since the three functions take the value 1 in $r = R$, if $\nu (r)$ has a lateral derivative $\nu _+'(R)$, one necessarily has
  $$ \varPsi _ +'(R) = - \frac{1}{2R}\frac{1 + k}{1-k} \le \nu _+'(R) \le \varPsi _+'(R ) =- \frac{1}{2R}\frac{1 - k}{1+k} < 0. $$
  Therefore, such a solution cannot has a differentiable extension to $(0, \infty )$ since such a extension would reach its maximum at $r = R$ and thus its derivative should be 0 at this point.

$$\square $$

### Theorem 2

Suppose that $W(r) = [\mu (r),\nu (r)]^T$ is a smooth stationary solution defined in a neighborhood of $r = R$ that satisfies (41) with $C_1 < 0$. Then:

1. $$\displaystyle \nu (R) = \frac{A-1}{A+ 1}$$, with $$\displaystyle A = \left( -\frac{C_1}{R^2} \right) ^{\frac{2K^2}{1 - k^2}}.$$
2. *W*(*r*) is supersonic for $r \not = R$.

### Proof

1. According to Cases 2 and 3, the only possibility to build a continuous stationary solution at $r = R$ is to take $\nu (r) = \nu _{sup}$ for $r < R$ and
  $$ \lim _{r \rightarrow R^-} \nu (r) = \frac{A-1}{A + 1}, \quad A = \left( -\frac{C_1}{R^2} \right) ^{\frac{2K^2}{1 - k^2}}.$$
2. If $\nu (r) = \nu _{sub}$ for $r > R$ one has
  $$\begin{aligned} \nu (r) \in \left[ \frac{k-B}{KB -1}, B\right) , \end{aligned}$$
  so that
  $$\begin{aligned} \lim _{r \rightarrow R^+} \nu (r) = 1 \not = A, \end{aligned}$$
  and $\nu$ would be then discontinuous at $r = R$. The only possibility is then to take $\nu (r) = \nu _{sup}$ for $r > R$.

$$\square $$

### Theorem 3

Suppose that $W(r) = [\mu (r),\nu (r)]^T$ is a transonic continuous stationary solution defined in a neighborhood of $r = R$ such that (i) *W* is differentiable for $r \not = R$ and has lateral derivatives at $r = R$; (ii) *W*(*r*) is supersonic for $r < R$; (iii) *W*(*r*) is subsonic for $r > R$. Then, necessarily:

1. $$C_1 \ge 0$$, $$C_2 = 0$$.
2. *W* is not differentiable at $r = R$.

### Proof

From the proof of Theorem () we know that it is not possible to build continuous transonic stationary solution if $C_1 < 0$. Therefore, $C_1 \ge 0$. On the other hand, from the proof of Theorem () we know that

$$ \varPsi _ +'(R) = - \frac{1}{2R}\frac{1 + k}{1-k} \le \nu _+'(R) \le \varPsi _+'(R ) =- \frac{1}{2R}\frac{1 - k}{1+k} < 0. $$

Therefore, such a solution cannot be differentiable at $r = R$ since it reaches its maximum at this point. On the other hand, since

$$ \mu = C_2 \frac{1 - \nu ^2}{r^2 (\nu - B)(1 - B\nu )}$$

an easy application of L’Hopital’s rule shows that

$$\begin{aligned} \lim _{r \rightarrow R^+} \mu (r) = \infty \end{aligned}$$

unless $$C_2 = 0$$. $$\square $$
